# Pan-cancer analysis reveals MTTP as a prognostic and immunotherapeutic biomarker in human tumors

**DOI:** 10.3389/fimmu.2025.1549965

**Published:** 2025-03-27

**Authors:** Wenjia Wang, Yaping Gao, Yihan Liu, Shurui Xia, Jiayao Xu, Liwei Qin, Yongxu Jia, Yanru Qin

**Affiliations:** Department of Oncology, The First Affiliated Hospital of Zhengzhou University, Zhengzhou, China

**Keywords:** MTTP, pan-cancer, prognosis, tumor microenvironment, gastric cancer

## Abstract

**Introduction:**

Microsomal triglyceride transfer protein (MTTP) is an essential lipid transfer protein for the synthesis and secretion of very low density lipoprotein (VLDL) in hepatocytes and chylomicrons (CM) in intestinal cells. Further researches have revealed that MTTP exerted its functions in a variety of tissues beyond the liver and intestine, including the heart, neural tissues and antigen-presenting cells. Dysregulation of MTTP expression can lead to many diseases, such as lipid metabolism disorders, insulin resistance and cardiovascular diseases. Despite its importance, research on MTTP in cancer is limited, with no comprehensive pan-cancer studies available.

**Methods:**

MTTP expression was explored with the TIMER 2.0 and Sangerbox databases. The pathological stages and survival analysis of MTTP were analyzed via GEPIA and Kaplan Meier plotter. The gene mutations of MTTP were analyzed by cBioPortal database. The immune landscape of MTTP in the tumor microenvironment(TME) was analyzed using the TIMER 2.0 and single-cell sequencing. Based on the RNA-seq data in TCGA, we constructed GSEA enrichment analysis for MTTP. We identified the pro-tumor and anti-ferroptosis functions of MTTP in gastric cancer (GC) cells by *in vitro* and *in vivo* experiments, and analyzed the effect of TME on ferroptosis by single-cell sequencing.

**Results:**

MTTP expression was elevated in at least 1/3 tumors. High expression of MTTP was associated with poor prognosis in most tumors. MTTP levels were significantly correlated with three scores (immune, stromal, and extimate) and immune checkpoints in at least half of tumor types. Single cell sequencing of immune cells showed that MTTP was mainly expressed in macrophages, especially in microglia. MTTP increased in GC and MTTP knockdown limited the proliferation, migration and invasion abilities of GC cells, accompanied by increased sensitivity to ferroptosis. In addition, analyzing the ferroptosis genes associated with MTTP at the single cell level, we found that macrophages may be involved in the ferroptosis process in GC.

**Conclusions:**

Our pan-cancer study emphasizes that MTTP is a promising prognostic and immunotherapeutic biomarker in tumors. High expression of MTTP is correlated with the infiltration of diverse immune cells and regulates ferroptosis in GC cells, providing a potential target for tumor immunotherapy.

## 1 Introduction

Cancer cells perturb lipid metabolic pathways and subsequently promote multiple tumorigenic functions and dysregulation of cellular metabolism is a hallmark of cancer cells (1). In contrast to normal cells, which mainly obtain the bulk of the required lipids from the circulation, cancer cells are known to synthesize a substantial fraction of their lipids *de novo (* 2, 3). Increased *de novo* lipogenesis, mediated by upregulation of essential metabolic enzymes, is considered a universal hallmark of human tumors (4, 5). Evidence is also growing that lipids play a key modulatory role in crosstalk between tumor and tumor microenvironment (TME), metastatic melanoma cells, while traversing the oleate-rich lymphatic environment, utilize oleate to protect themselves from ferroptosis (6). The rewiring of lipid metabolism in cancer holds potential for the development and use of diagnostic or prognostic biomarkers, and multiple enzymes involved in lipid metabolism are also potential targets for therapy. Microsomal triglyceride transfer protein (MTTP) is an endoplasmic reticulum resident protein, which plays important roles in triglyceride(TG) transport, as well as very low density lipoprotein (VLDL) assembly, secretion and lipid droplet maturation (7–9). MTTP is highly expressed in the intestine and liver, the two major sites of the synthesis and secretion of triglyceride-rich lipoproteins (10). The kidney and heart rank as the third and fourth most expressive organs for MTTP, respectively. Nonetheless, the mRNA levels of MTTP in these tissues are only 3-5% of those found in the liver (11). Beyond the above mentioned tissues, current researches have dentified the presence of MTTP in a multitude of tissues, such as retina, neurons and dipocytes (12, 13). Dougan et al. (14) showed that MTTP was expressed in various antigen presenting cells, such as monocytes, B cells, splenocytes, bone marrow and monocyte derived dendritic cells and MTTP could promote CD1-mediated lipid antigen presentation in these cells. These studies indicate that MTTP plays an essential role in sustaining the normal physiological functions in human body.

Several studies have confirmed that MTTP variant affected hepatic steatosis and plasma lipid, leading to systemic metabolic dysfunction (15, 16). Pluripotent stem cell derived cardiomyocytes carrying an MTTP mutation displayed impaired APOB secretion, lipid accumulation, and increased sensitivity to cellular stress (17). Emerging evidence points to an association of MTTP with tumorigenesis, diethylnitrosamine-treated Mttp-KO mice exhibited hepatic steatosis with increased tumor burden compared with flox controls (18), suggesting that the loss of MTTP function may lead to the occurrence of tumor. However, several studies have indeed confirmed that after the formation of tumors, inhibiting MTTP could slow the progression of cancer. For instance, pharmacologic inhibition of the MTTP could attenuate dyslipidemia and reduce tumor growth in tumor-bearing hyperlipidemic obese mice, which was related to MTTP-mediated altered circulating lipids (19). In advanced colorectal cancer (CRC) patients with a high body fat ratio, the expression of MTTP in plasma exosomes was increased, which served as an inhibitor of ferroptosis and reduced sensitivity to chemotherapy in tumor lesion (20). In brain tumors, MTTP expression was significantly upregulated, and increased MTTP expression was associated with poor patient survival (21). Loss of MTTP slowed gliomagenesis and increased the tumor triacylglycerides in mouse (22).

Using public databases, pan-cancer analysis can most effectively understand the molecular mechanisms and predictive value of genes in tumor biology, thereby maximizing clinical diagnosis and treatment options. Therefore, our study, for the first time, used public databases such as TCGA and GTEx to conduct a pan-cancer analysis of MTTP and explored its impact on cancer prognosis. In addition, to further analyze the relationship between MTTP and tumor immunity, this study evaluated the correlation between MTTP expression and immune checkpoint related genes and immune cell infiltration scores. Based on the results of pan-cancer analysis, we found that MTTP was closely related to the prognosis of GC. Therefore, we further verified the effect of MTTP on the malignant biological function of GC by comprehensive multi-omics analysis and *in vivo* and *in vitro* combination experiments.

Taken together, our findings provide new insights into the commonalities and differences of MTTP in pan-cancer, reveal the role of MTTP in tumor immunity, and demonstrate the potential prognostic significance and therapeutic role of MTTP in GC.

## 2 Materials and methods

### 2.1 Gene expression and survival prognosis analysis

We used the “Gene_DE” function of the Tumor Immune Estimation Resource, version 2 (TIMER2.0, http://timer.cistrome.org/) website to observe the expression of MTTP in tumor tissues and corresponding normal tissues. For tumors without corresponding normal tissues or that had a limited number of corresponding normal tissue samples, we used the Sangerbox website (http://www.sangerbox.com/), which matched TCGA and normal Genotype-Tissue Expression (GTEx) data. We used the Human Protein Atlas (HPA, https://www.proteinatlas.org/), mRNA expression information of MTTP in various tissues and cancer cell lines was found in the “TISSUE” and “CELL LINE” modules, respectively. In addition, we analyzed the relationship between MTTP expression and different pathological stages of tumors in the TCGA database using the “Stage Plot” module on the Gene Expression Profiling Interactive Analysis (GEPIA2) website. We used the “Survival Map” module of the GEPIA2 tool to obtain significance map data for overall survival (OS) and disease free survival (DFS) for all TCGA tumors. Survival curves were analyzed using the “survival analysis” module of GEPIA 2. In addition, we used Kaplan Meier plotter web (https://kmplot.com/analysis/) to analyze the effects of MTTP expression on OS and RFS (Relapse-free survival) of breast cancer, ovarian cancer, lung cancer, pancreatic cancer, AML and myeloma.

### 2.2 Genetic alteration analysis

We selected “TCGA Pan Cancer Atlas Studies” in the “Quick select” section of the cBioPortal website (https://www.cbioportal.org/) to analyze the genetic alterations of MTTP in TCGA tumors. We observed the frequency of MTTP alterations and mutation types in the”Cancer Types Summary” module. In addition, the “Mutations” module provides information on the mutation sites of MTTP and the 3D structure of the protein. We also analyzed the survival information of tumor patients with MTTP gene alterations in the “Comparison/Survival” module and generated survival maps. We used the “TCGAplot” R package to draw the radar chart of the correlation between MTTP expression and TMB, and MSI (23).

### 2.3 Immune cell infiltration analysis

First, we explored the relationship between MTTP expression and immune infiltration in TCGA tumors using the “Immune” module on the TIMER2.0 website. Cancer-associated fibroblasts (CAFs), NKT cells and CD8^+^ T cells were targeted for evaluation of immune infiltration using a suite of analytical tools, including the TIMER, EPIC, MCPCOUNTER, CIBERSORT, CIBERSORT-ABS, XCELL, TIDE, and QUANTISEO algorithms. The correlation of MTTP expression with immune-related genes, including those encoding chemokine receptor proteins, chemokine, immune stimulators and immune inhibitors was also analyzed. The relationships between MTTP expression and the three immune scores (StromalScore, ImmuneScore and EstimateScore), tumor infiltration immune cells (TIICs) in multiple tumors were explored.

### 2.4 Single-cell RNA-sequencing dataset download and analysis

The expression matrix and metadata-information file of the single-cell RNA-sequencing dataset GSE136001,GSE140228, GSE234129 and GSE183904 were retrieved from the Gene Expression Omnibus (GEO: https://www.ncbi.nlm.nih.gov/geo/) database. In GSE183904, 15 GC samples were selected for analysis. The “Seurat” R package was employed to conduct a comprehensive analysis of single-cell sequencing data (24). This analysis encompassed several critical steps: object construction, data standardization, dimensionality reduction, clustering, and marker gene identification. The CreateSeuratObject function was utilized to establish a seurat object, with initial filtering criteria set to a minimum of 3 cells, 200 features, thereby conducting preliminary gene and cell filtration. Subsequent filtering thresholds were established to refine cell selection: the feature count ranged between 200 and 6000, the count threshold was set between 300 and 40,000, the overexpression double cutoff was set at 0.15, mitochondrial gene expression was capped below 30%, and red blood cell read percentage was restricted to less than 20%. The top 2000 hypervariable genes were selected to serve as inputs for principal component analysis (PCA). The first 15 principal components (PCs) were identified as pivotal for subsequent analysis using the ElbowPlot function. The single-cell sequencing data originated from multiple datasets, necessitating the use of the “harmony” R package for batch correction to mitigate batch effects that could confound downstream analysis. Following debugging and referencing the clustering outcomes against the original contributions, the FindClusters algorithm was deployed to discern tumor cell subsets with a resolution parameter of 0.5, the data’s dimensionality was further condensed using t-distributed Stochastic Neighbor Embedding (t-SNE). Cell clusters were annotated based on experimental metadata references, designating cell types such as T cells, B cells, epithelial cells, macrophages, monocytes, fibroblasts, endothelial cells, mast cells, etc.

### 2.5 MTTP-related gene enrichment analysis

We first searched the STRING website (https://string-db.org/) using the query of a single protein name “MTTP”. Subsequently, we set the following main parameters: minimum required interaction score [“Low confidence (0.150)”], max number of interactors to show (“no more than 50 interactors” in 1st shell). Finally, the available experimentally determined MTTP-binding proteins were obtained. We used the “Similar Gene Detection” module of GEPIA2 to obtain the top 100 MTTP-correlated targeting genes. We combined the two sets of data to perform KEGG(Kyoto encyclopedia of genes and genomes) pathway analysis and GO(Gene ontology) enrichment analysis. For GSEA enrichment analysis, we used the “limma” R package to analyze the differentially expressed genes(DEGs) between patients with high and low MTTP expression, and then sorted them in descending order according to the logFC of gene expression. The Hallmark pathway scores were obtained for all cancers using the “GSEABase” and “clusterProfiler” R package.

### 2.6 Cell culture

Immortalized gastric epithelial cell line GES-1 and seven GC cell lines MKN28, MGC803, BGC823, HGC27, SNU-1, AGS and MKN45 were obtained from Chinese academy of Sciences (Shanghai, China). Cell lines were cultured in RPMI-1640 medium (Gibco, China), with 10% fetal bovine serum (Gibco, China) and were grown in an atmosphere of 5% CO2 at 37°C.

### 2.7 Plasmids and antibodies

MTTP short hairpin RNAs (shRNAs) plasmid and its control plasmid were purchased from GeneCopoeia. The following antibodies were used in our study: MTTP (1:1000) (Affinity Biosciences DF6591), ACSL4 (1:2000) (proteintech 22401-1-AP), GPX4 (1:2000) (proteintech 67763-1-Ig), SCL7A11 (1:2000) (abcam ab307601), b-actin (1:5000) (proteintech 66009-1-Ig).

### 2.8 IHC analysis

We collected six pairs of primary cancerous and non-cancerous tissues obtained from surgery for GC from the pathology bank of the first affiliated hospital of Zhengzhou university, and it was confirmed that these patients had never received radiotherapy or chemotherapy prior to surgery. IHC staining was performed using the standard streptavidin–biotin–peroxidase complex method as described previously (25). An immunoreactivity score system was applied in the analysis of IHC staining. The percentage of positive cells was scored as follows: 0, <5%, 1, 5–25%, 2, 25–50%, 3, 50–75%, 4, 75–100%. The intensity of staining was scored as follows: 0, negative; 1, weak; 2, moderate; 3, strong. The total score was determined by the following formula: staining index=positive percentage × intensity.

### 2.9 *In vitro* functional assays

Cell viability was assayed by the CCK-8 reagent (C6005M, UElandy) following with the manufacturer’s protocol. In brief, GC cells (1 × 10^3^ cells/well) were transferred into a 96-well plate. Two duplicate wells were set for each group with a negative control. 10 μL of the CCK-8 reagent was then added into each well with a total volume of 100 μL medium, the plates were incubated at 37°C for 2 h, and the OD values were measured with microplate reader at a wavelength of 480 nm. CCK8 were performed once a day until day 6. For colony formation assay, 1 × 10^3^ cells were plated in 6-well plate. Surviving colonies (>50 cells per colony) were stained with 0.05% crystal violet and counted after one week in culture. For wound healing assay, the confluent monolayer of cells was scratched with a fine pipette tip, and cell migration into the wound was observed after 48 h by microscopy. Cell migration assays were performed using a 24-well transwell chamber (Coring, USA) according to the manufacturer’s instructions. Briefly, cells (8 × 10^4^) in 1640 medium with FBS free were layered in the upper chamber, and medium containing 10% FBS was applied to the lower chamber. The chambers were then incubated for 24 h for cell migration at 37°C. After removing the cells in the upper surface of filter with cotton swab, the invasive cells attached to the lower surface of the membrane were fixed with 4% paraformaldehyde solution, stained with 0.05% crystal violet and then quantified by counting the cell number at ten random fields under a microscope.

### 2.10 Animal experiments

Animals were housed in a pathogen-free facility, and the animal experiments were performed according to the guidelines approved by the Zhengzhou University Institutional Animal Care and Use Committee. 2×10^6^ AGS shMTTP or control cells were injected into axilla of 4-week-old female NU/NU mice (Beijing Vital River Laboratory Animal Technology Co., Ltd.) (n = 5 per group). After 18 days, the mice were euthanized. The xenograft tumors were weighed.

### 2.11 Measurement of MDA, lipid ROS and mitochondrial membrane potential

The MDA level of cells were measured by Malondialdehyde(MDA) Content Assay Kit (BC0025, Solarbio). Lipid reactive oxygen species (ROS) generation was measured by adding 5μM BODIPY™ 581/591 C11 (a lipid peroxidation sensor, D3861; Thermo Fisher Scientific) for 30min 37°C. Oxidized forms of cells were confirmed by fluorescence microscope. For the detection of MMP, cells were incubated with 500ul of JC-1 staining working solution for 20 min at 37°C (40706ES60, Yeasen). The fluorescent images were captured using a fluorescence microscope.

### 2.12 Statistics analysis

We performed all statistical analyses using the R Project software (https://www.r-project.org/, version 4.3.1) and GraphPad Prism 8 (GraphPad, Inc., CA, USA) software. Differences between two groups were calculated using the paired two-tailed Student’s t-test or the Mann–Whitney–Wilcoxon test, Comparisons among three groups were performed using ANOVA or the Kruskal–Wallis rank-sum test. Spearman’s rank correlation analysis computed correlation coefficients. Kaplan–Meier survival analysis with the log-rank test compared the prognosis between two subgroups, and the hazard ratio (HR) of variables was calculated using univariate and multivariate Cox proportional hazard regression analyses. Statistical significance was denoted as follows: *, P<0.05; **, P <0.01; ***, P <0.001; ****, P <0.0001; ns: not significant.

## 3 Results

### 3.1 Gene expression and survival prognosis analysis of MTTP in pan-cancer

We first analyzed the expression pattern of MTTP in different normal tissues and cancer cell lines. As shown in **Supplementary Figure 1**, according to Consensus, HPA, GTEx, and Function Annotation of the Mammalian Genome 5 (FANTOM5) datasets, MTTP was highly expressed in the small intestine and liver, showing higher mRNA tissue specificity. In addition, MTTP was highly expressed in liver cancer and GC cells in the HPA dataset (**Supplementary Figure 2**).

**Figure 1:**
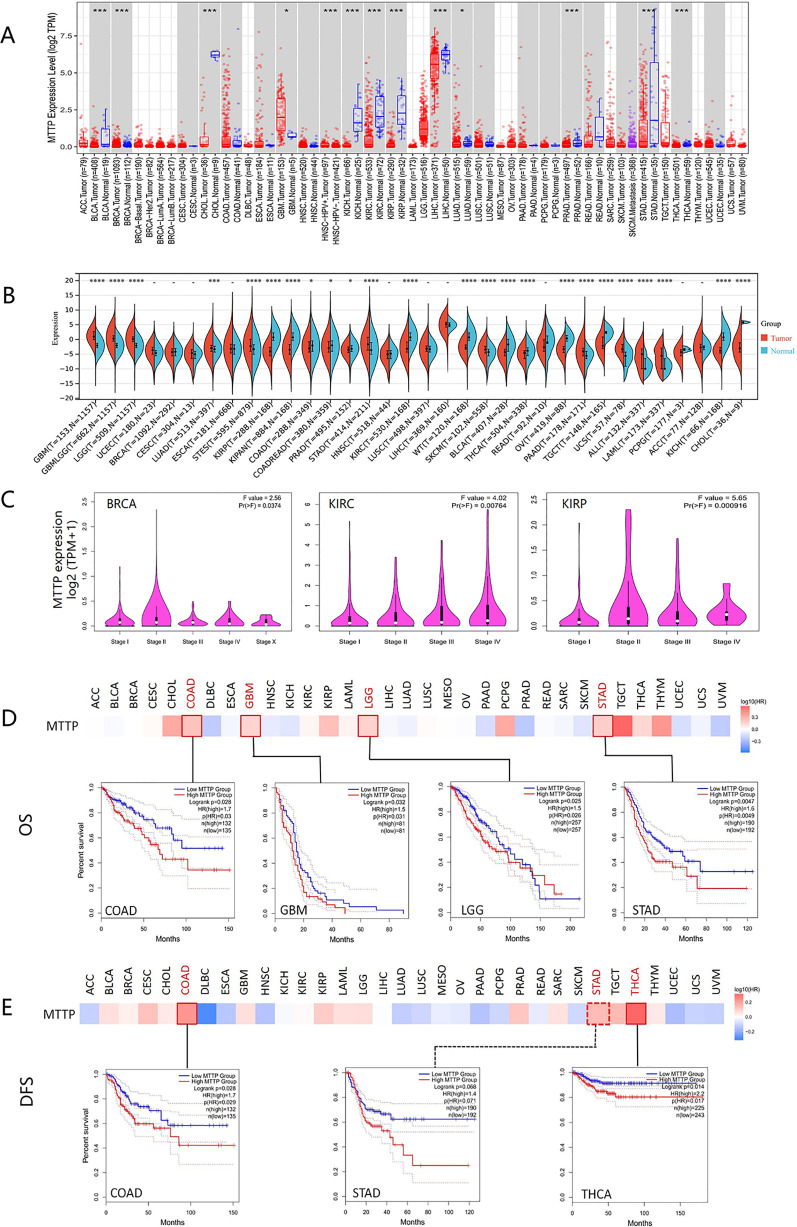
Expression profiles and survival analysis of MTTP in pan-cancer. **(A)** MTTP mRNA expression levels in different tumors and corresponding normal tissues from TCGA database by TIMER2. **(B)** MTTP mRNA expression levels in different tumors and corresponding normal tissues from TCGA and GTEx database by SangerBox. **(C)** The relationship between MTTP and the pathological stage of BRCA, KIRC and KIRP was analyzed based on TCGA data. **(D)** Overall survival analysis in patients with different MTTP expression levels. **(E)** Disease-free survival analysis inpatients with different MTTP expression levels *, P <0.05; ***, P <0.001; ****, P <0.0001.

We applied the TIMER2 approach to analyze the expression status of MTTP across various cancer types of TCGA. As shown in **Figure 1A**, MTTP was highly expressed in glioblastoma multiforme (GBM) and poorly expressed in bladder urothelial carcinoma (BLCA), breast invasive carcinoma (BRCA), cholangio carcinoma (CHOL), kidney chromophobe (KICH), kidney renal clear cell carcinoma (KIRC), kidney renal papillary cell carcinoma (KIRP), liver hepatocellular carcinoma (LIHC), lung adenocarcinoma (LUAD), prostate adenocarcinoma (PRAD), stomach adenocarcinoma (STAD), thyroid carcinoma (THCA)(all P<0.05). After including the normal tissue of the GTEx dataset as controls based Sangerbox database, we further evaluated the expression difference of MTTP between the normal tissues and tumor tissues(**Figure 1B**). The expression level of MTTP in the tumor tissues of GBM, STAD, brain lower grade glioma (LGG), LUAD, stomach and Esophageal carcinoma (STES), skin cutaneous melanoma (SKCM), pancreatic adenocarcinoma (PAAD), uterine carcinosarcoma (UCS), acute lymphoblastic leukemia (ALL), acute myeloid leukemia (LAML) is higher than the corresponding control tissues (P<0.05). Conversely, the expression level of MTTP in KIRP, PRAD, KIRC, BLCA, THCA, KICH, CHOL, pan-kidney cohort (KIPAN), colon adenocarcinoma (CDAD), high-risk wilms tumor (WT), ovarian serous cystadenocarcinoma (OV) and testicular germ cell tumors (TGCT) is lower than the corresponding control tissues (P<0.05). We then used the GEPIA2 website to analyze the correlation between MTTP expression and the tumor pathological stage and observed changes in MTTP expression at different stages of BRCA, KIRC and KIRP (**Figure 1C**). However, no significant correlation was observed for other tumors.

We divided the cancer cases into high-expression and low-expression groups according to the expression levels of MTTP and investigated the correlation of MTTP expression with the prognosis in different tumors, mainly using the datasets of GEPIA2. As shown in **Figure 1D**, highly expressed MTTP was linked to poor prognosis of overall survival (OS) for cancers of COAD (P = 0.028), GBM (P = 0.032), LGG (P = 0.025), and STAD (P = 0.0047). Disease-free survival (DFS) analysis data showed a correlation between high MTTP expression and poor prognosis in COAD (P = 0.028) and THCA (P = 0.014). In addition, the DFS curves of STAD were slightly statistically different (P=0.068) (**Figure 1E**).

Moreover, we used the Kaplan-Meier plotter tool to analyze the effects of MTTP expression on OS and RFS (Relapse-free survival) of OV, BRCA, LUAD, LUSC, PAAD, Myeloma and AML (**Supplementary Figure 3**). The result presented the patients with higher expression MTTP had a better OS for OV(P = 0.015) and a better RFS for OV and BRCA(P<0.001). In contrast, the patients with higher expression MTTP had a poor OS for Myeloma (P<0.001) and AML (P = 0.0016), and a poor RFS for Myeloma and LUAD (P<0.001). The expression level of MTTP was not correlated with OS and RFS in LUSC and PAAD.

### 3.2 Genetic alteration analysis

**Figure 2:**
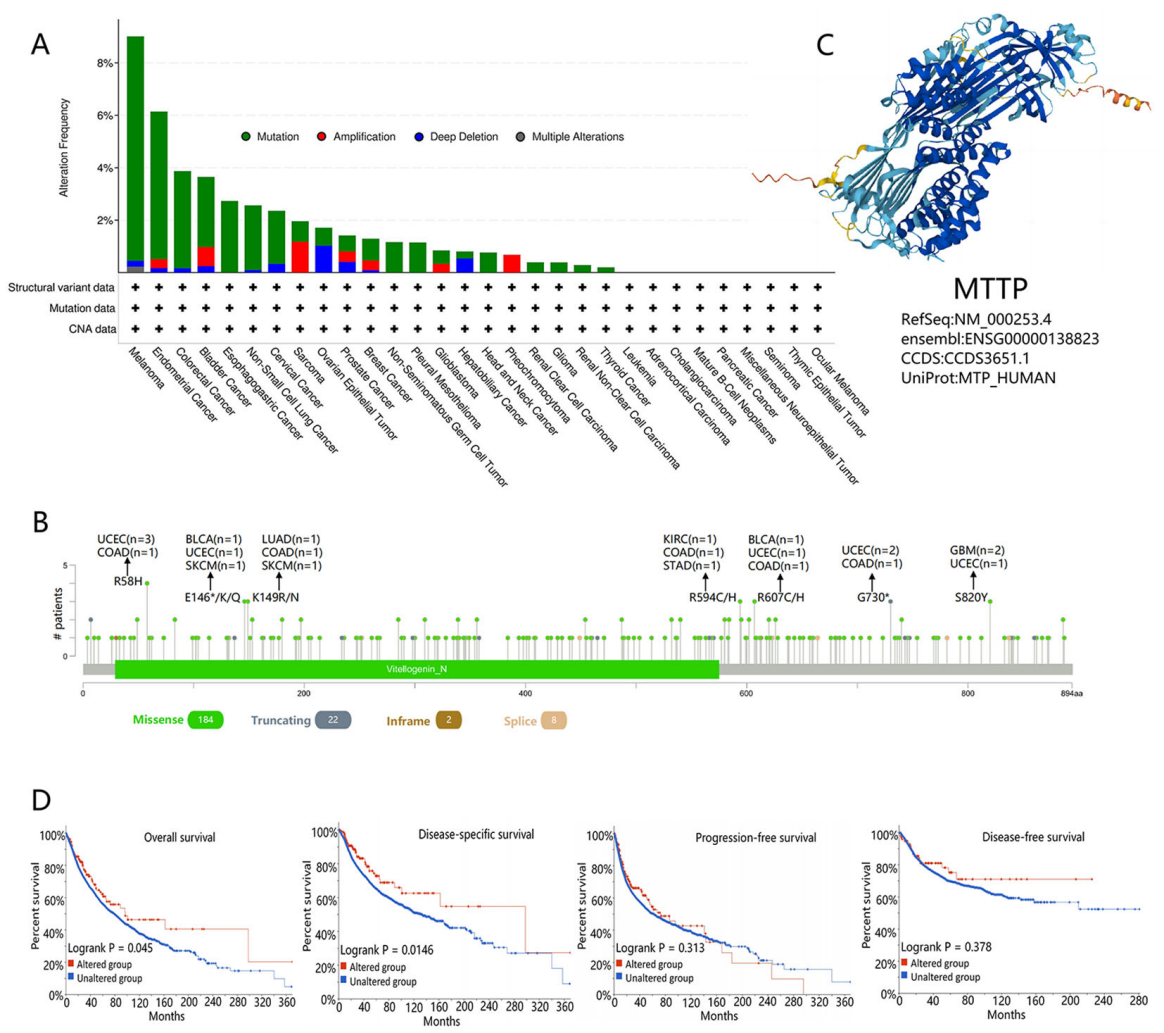
Mutational characteristics of MTTP in TCGA tumors based the cBioPortal tool. **(A)** The frequency of the mutation type of MTTP in pan-cancer. **(B)** The mutation site alterations of MTTP. **(C)** The 3D protein structure of MTTP. **(D)** The potential correlation between mutations in MTTP and overall, disease-specific, progression-free, and disease-free survival of all TCGA tumors.

Genetic alterations play a key role in tumorigenesis, and we observed the genetic alterations of MTTP in TCGA PanCancers Atlas Studies through the cBioPortal database. As shown in **Figure 2A**, the highest alteration frequency of MTTP (9.01%) appeared for patients with SKCM with “mutation” as the primary type. The “amplification” type was the primary type in the Sarcoma, which showed an alteration frequency of 1.96%. The “deep deletion” type was the primary type in patients with OV, which showed an alteration frequency of 1.71%. The types, sites and case number of the MTTP genetic alteration were further presented in **Figure 2B**. We found that missense mutation of MTTP was the main type of genetic alteration. **Figure 2C** showed the protein structure of MTTP. Additionally, we explored the potential association between genetic alteration of MTTP and the clinical survival prognosis of all tumor patients. As shown in **Figure 2D**, compared with patients without MTTP mutation, the MTTP mutation in patients had between OS (P =0.045), DSS (P =0.0146), however, there was no difference in PFS and DFS between patients with and without the MTTP mutation.

### 3.3 Immune infiltration analysis

**Figure 3:**
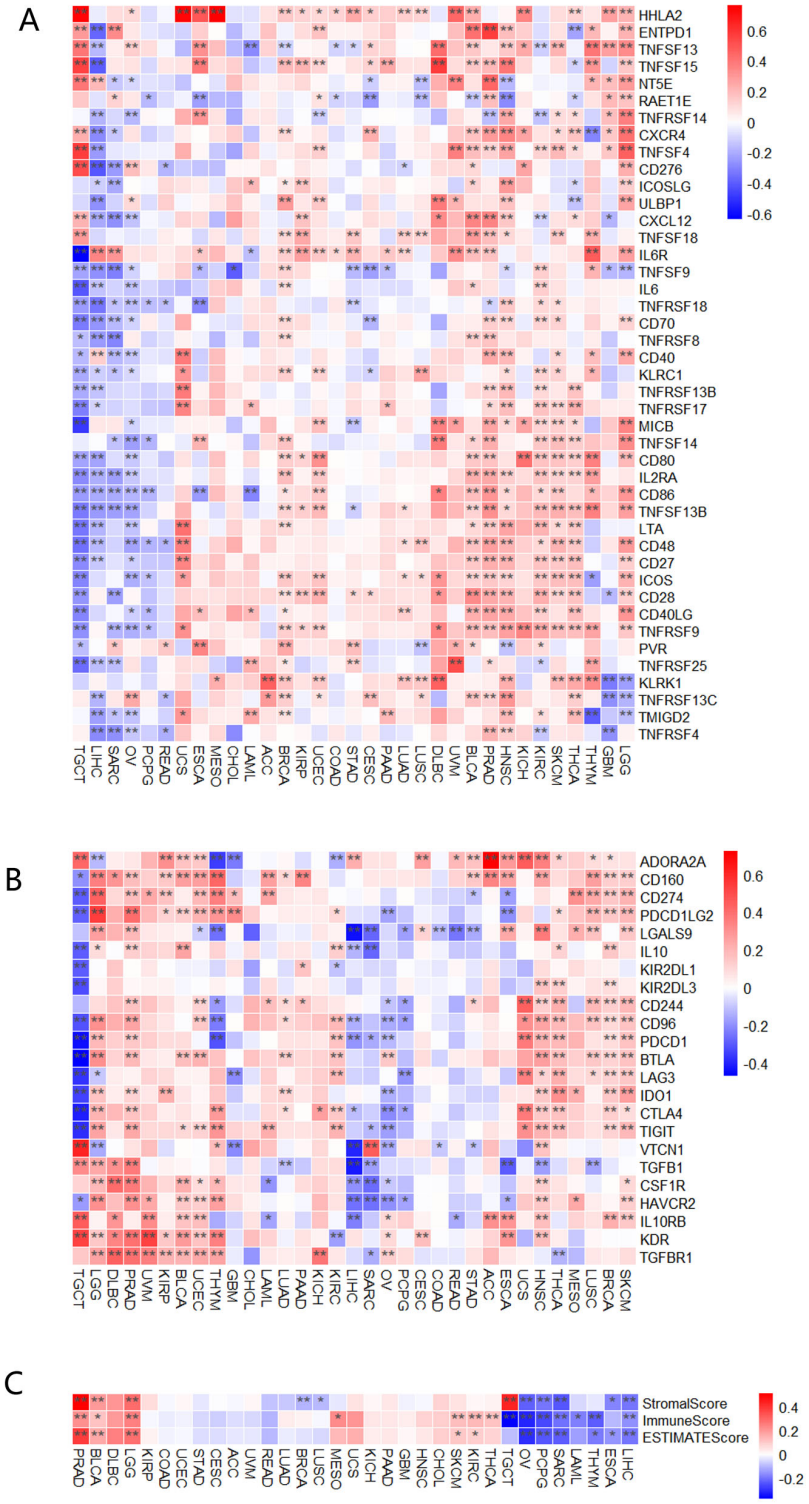
Correlations between MTTP expression and immune checkpoints genes, three immune scores. **(A)** Correlations between MTTP expression and immune stimulators genes in different tumors. **(B)** Correlations between MTTP expression and immune inhibitors genes in different tumors. **(C)** Correlations between MTTP expression and three immune infiltration scores in different tumors *, P <0.05; **, P <0.01.

To explore the role of MTTP in the immune regulation and immune response of the TME, we initially conducted gene co-expression analysis to investigate the relationship between MTTP expression and immune-related genes in pan-cancer. MTTP expression was positively correlated with most immune stimulators (**Figure 3A**), immune inhibitors (**Figure 3B**), chemokine receptors (**Supplementary Figure 4A**), and chemokines (**Supplementary Figure 4B**) across most tumor types. In these immune-related genes, HHLA2, CD160, PD-L1 and PDCD1LG2 (PD-L2) were significantly positively correlated with MTTP expression in most tumor types. In addition, we also observed that most immunosuppressive genes were positively correlated with MTTP expression in LGG, PRAD, BLCA, UCEC, HNSC, BRCA and SKCM, and most immunosuppressive genes are negatively correlated with MTTP expression in TGCT and LIHC. We also evaluated the correlations between MTTP expression and the three immune scores (StromalScore, ImmuneScore and EstimateScore) (**Figure 3C**), MTTP expression in PRAD, BLCA, LGG, SKCM and KIRC was positively correlated with immune infiltration, but MTTP expression in OV, PCPG, SARC, THYM, ESCA and LIHC was negatively correlated with immune infiltration in at least two immune scores systems.

**Figure 4:**
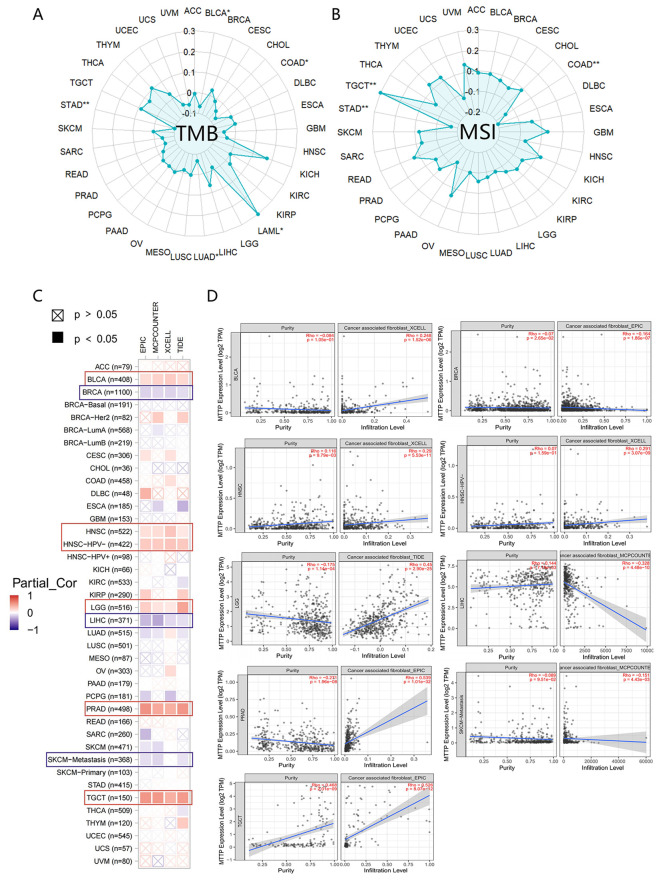
Correlations between MTTP expression and TMB, MSI and immune cell infiltration. **(A)** Correlations between MTTP expression and TMB. **(B)** Correlations between MTTP expression and MSI. **(C)** Correlation of MTTP expression in TCGA tumors with CAFs infiltration. **(D)** Scatter plots of correlation with statistical differences in tumors *, P <0.05; **, P <0.01.

We conducted an analysis to determine the correlation between MTTP expression levels and both tumor mutational burden (TMB) and microsatellite instability (MSI) (**Figures 4A, B**). MTTP expression in LAML was significantly and positively correlated with TMB, whereas MTTP expression in BLCA, COAD, LUAD, STAD was negatively correlated with TMB. We also found that MTTP expression was significantly positively correlated with the MSI of TGCT but negatively correlated with the MSI of COAD and STAD. We further used the TIMER, EPIC, MCPCOUNTER, CIBERSORT, CIBERSORT-ABS, XCELL, TIDE, and QUANTISEO algorithms to investigate the correlations between MTTP expression and tumor immune infiltration in different tumor types in TCGA. We observed a significant positive correlation between immune infiltration of CD8^+^ T cells and MTTP expression in BRCA, CESC, HNSC-HPV+, KIRC, LUAD, SKCM and THCA with all or most algorithms (**Supplementary Figures 5A, B**). In addition, our analysis revealed a statistically significant correlation between MTTP expression and NKT cells across 16 types of cancer, the correlation was positive in only 18.75% (3 out of 16), while it was negative in 81.25% (13 out of 16) (**Supplementary Figure 5C**). Furthermore, we observed that the expression of MTTP was significantly positively correlated with the infiltration of cancer-associated fibroblasts (CAFs) in BLCA, HNSC, HNSC-HPV-, LGG and PRAD but negatively correlated for BRCA, LIHC and SKCM-Metastasis (**Figures 4C, D**). Next, we plotted a clustering heatmap, which showed the correlations between immune cell infiltration and MTTP expression in 33 cancers. The results showed that a significantly positive correlation between MTTP and M2 macrophages in THYM, TGCT, UCEC, BLCA and LGG, but significantly negative correlation for THCA, SKCM, BRCA, SARC, ESCA and KIRC (**Supplementary Figure 4C**).

**Figure 5:**
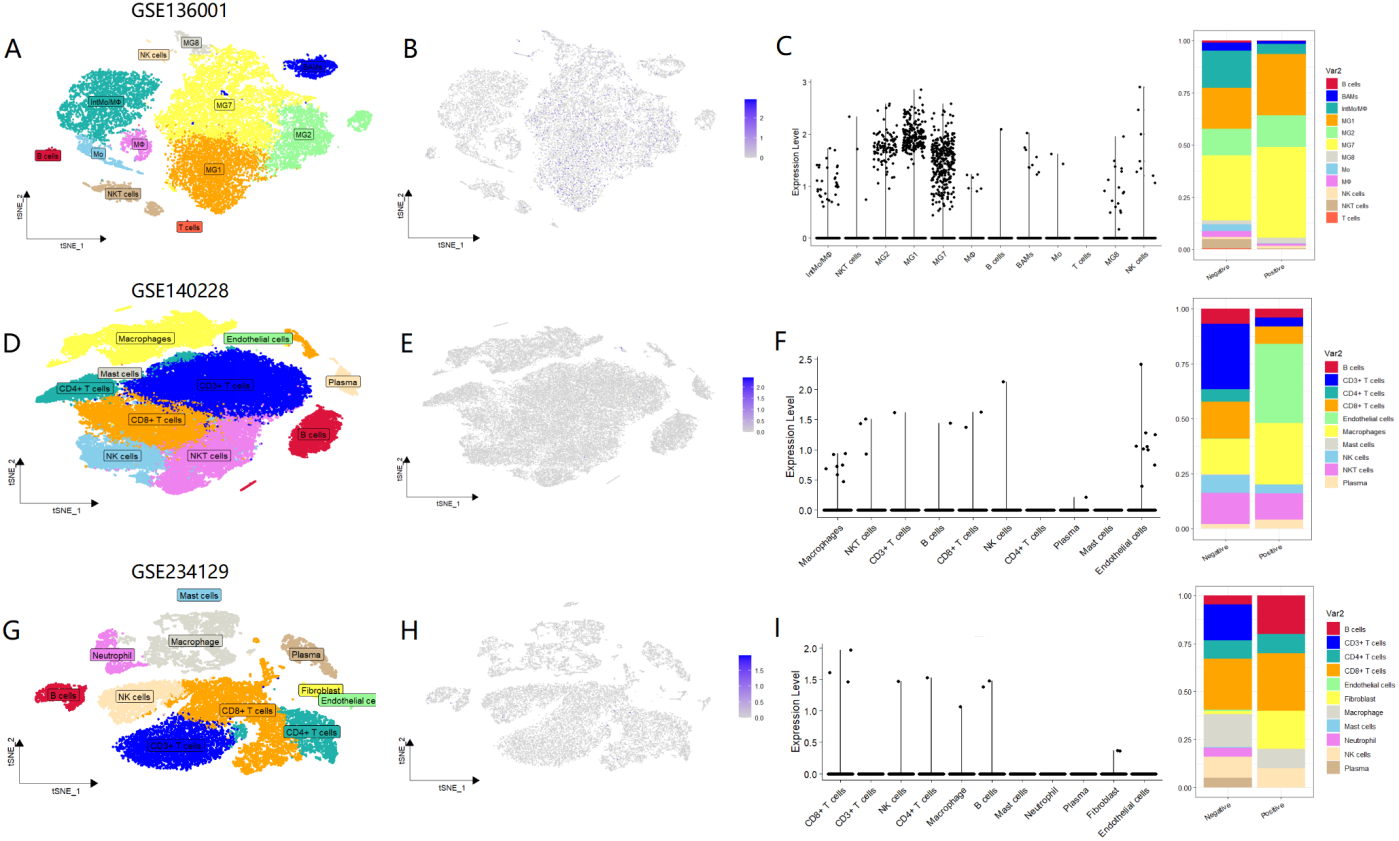
Expression of MTTP in three types of tumor immune cells. **(A)** t-SNE plot demonstrating cell clusters in GBM. Clusters annotations: MG: microglia, Mo: monocytes, IntMoMΦ: intermediate monocyte–macrophage, MΦ: macrophages, BAMs: CNS border-associated macrophages, NK cells: natural killer cells, NKT cells: natural killer T cells, B cells: B lymphocytes, T cells: T lymphocytes. **(B)** The expression profiles of MTTP in different immune cells in GBM. **(C)** The correlation between MTTP expression and the infiltration level of immune cells in GBM. **(D)** t-SNE plot demonstrating cell clusters in HCC. **(E)** The expression profiles of MTTP in different immune cells in HCC. **(F)** The correlation between MTTP expression and the infiltration level of immune cells in HCC. **(G)** t-SNE plot demonstrating cell clusters in GC. **(H)** The expression profiles of MTTP in different immune cells in GC. **(I)** The correlation between MTTP expression and the infiltration level of immune cells in GC.

Given that MTTP plays a role in the cell surface trafficking of CD1 in antigen-presenting cells (13), we conducted a further analysis of MTTP expression within immune cells. Firstly, we used scRNA sequencing to analyze the immune cellular landscape of GBM (**Figure 5A**, **Supplementary Figures 6A, B**), HCC (**Figure 5D**, **Supplementary Figures 6C, D**) and GC (**Figure 5G**, **Supplementary Figures 6E, F**). Immune cell composition in GBM notably differed from that of the two other solid tumors, a distinction primarily characterized by the presence of central nervous system-specific macrophages, known as microglia. Analysis using both violin plots and histograms revealed that MTTP expression was particularly high in microglia(MG1,MG2,MG7), surpassing that observed in bone marrow-derived macrophages(IntMo/MΦ) (**Figures 5B, C**). In HCC, MTTP was predominantly detected in macrophages, T cells, and endothelial cells (**Figures 5E, F**). However, the global expression levels of MTTP among immune cells was comparatively low in GC (**Figures 5H, I**).

### 3.4 GO and KEGG analysis of MTTP-related partners in pan-cancer

**Figure 6:**
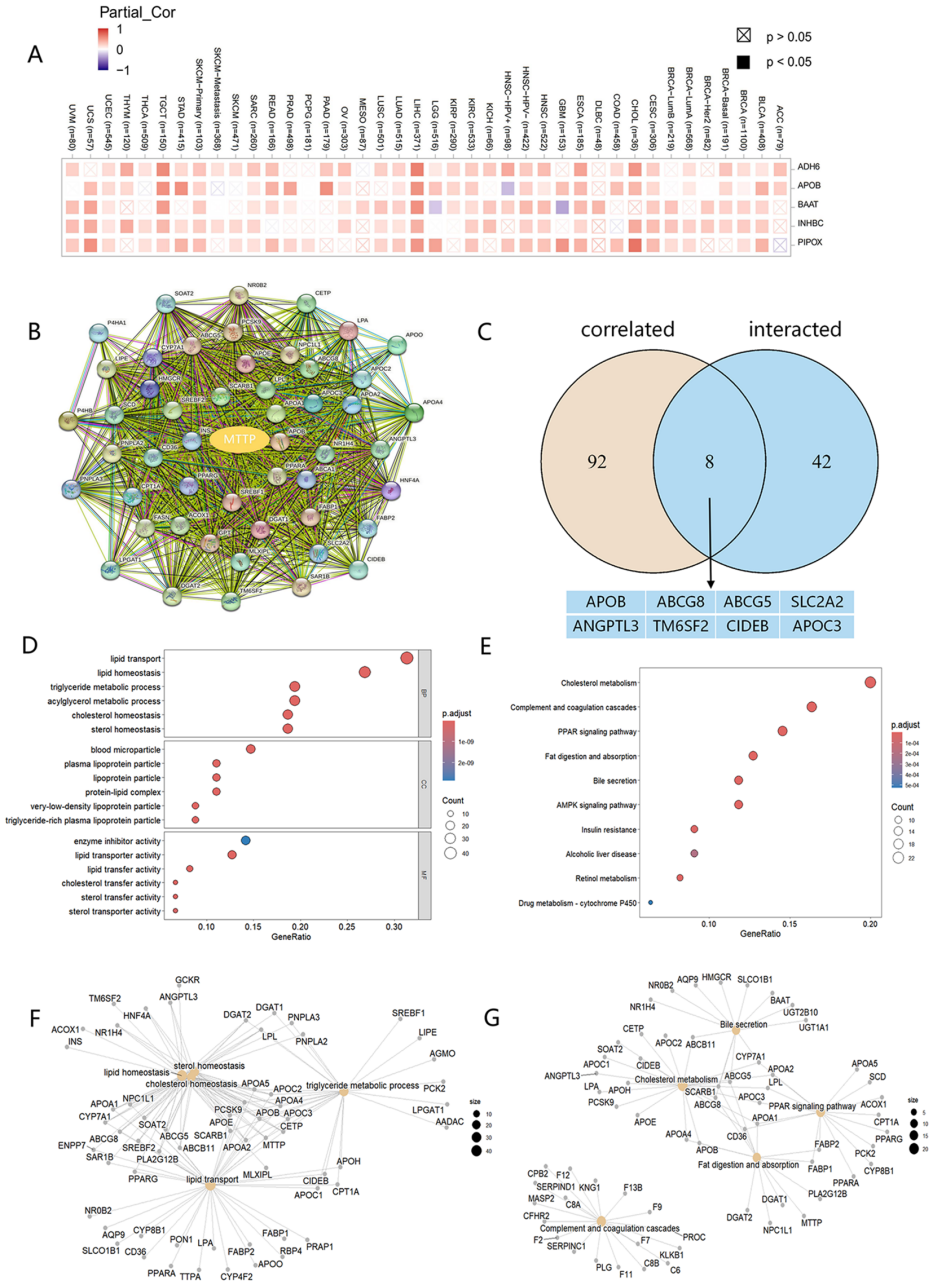
MTTP-related gene GO and KEGG enrichment analysis. **(A)** A heatmap of the correlations of MTTP expression with the expression of 5 potential target genes in TCGA tumors. **(B)** 50 experimentally validated MTTP-binding genes using the STRING tool. **(C)** An intersection analysis of the MTTP-binding and correlated genes. **(D)** GO enrichment analysis based on the MTTP-binding and correlated genes. **(E)** KEGG enrichment analysis based on the MTTP-binding and correlated genes. **(F)** The cnetplot of the GO analysis. **(G)** The cnetplot of the KEGG analysis.

To further analyze the molecular mechanism of MTTP development in tumors, we screened for MTTP-associated genes and MTTP-binding proteins. Based on the GEPIA2 website, we analyzed the tumor MTTP expression data in the TCGA dataset and obtained the top 100 genes associated with MTTP. The heatmap data showed a significant positive correlation between MTTP expression and five genes in the majority of detailed cancer types (**Figure 6A**). We obtained 50 MTTP-binding proteins supported by experimental evidence using the STRING website (**Figure 6B**). An intersection analysis of the above two datasets showed 8 common member, namely, APOB, ABCG8, ABCG5, SLC2A2, ANGPTL3, TM6SF2, CIDEB and APOC3 (**Figure 6C**). We further combined the two for Gene Ontology (GO) and Kyoto Encyclopedia of Genes and Genomes (KEGG) enrichment analysis. The GO data showed that the majority of MTTP-related genes were associated with lipid transport, lipid homeostasis and triglyceride metabolic process (**Figures 6D, F**). The KEGG data suggested that cholesterol metabolism, PPAR signaling pathway, Fat digestion and absorption, AMPK signaling pathway and insulin resistance may be related to the mechanism of MTTP in tumorigenesis (**Figures 6E, G**).

### 3.5 GSEA analysis of MTTP in different type of cancers

**Figure 7:**
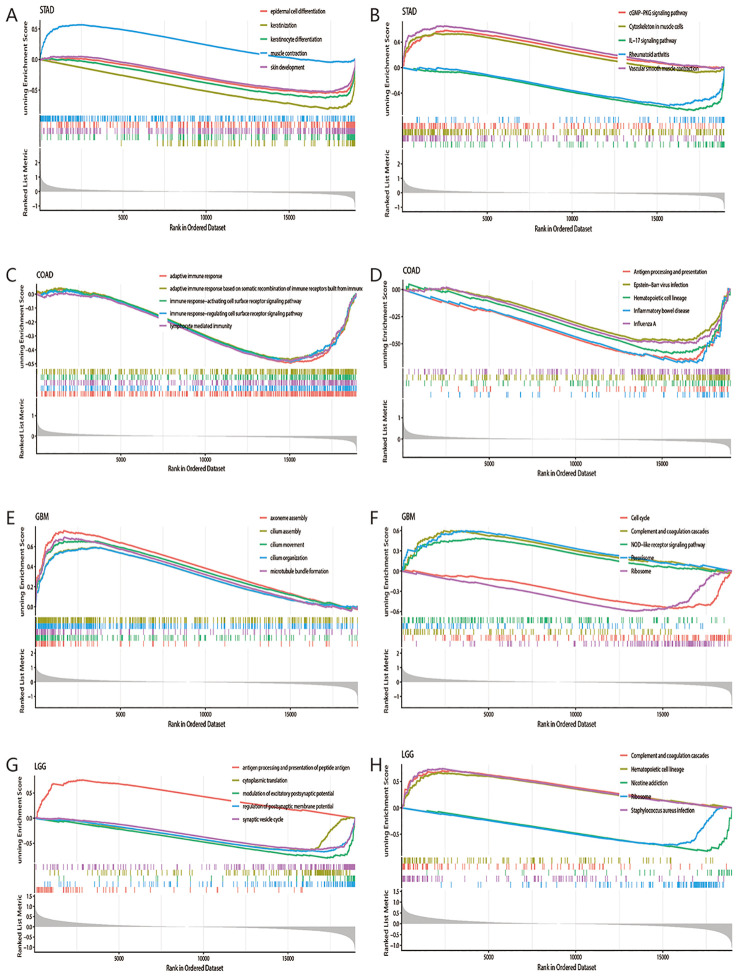
GSEA-GO and GSEA-KEGG analysis of the DEGs between MTTP high- and low-expression groups in four tumors. GSEA-GO **(A)** and GSEA-KEGG **(B)** analysis in STAD. GSEA-GO **(C)** and GSEA-KEGG **(D)** analysis in COAD. GSEA-GO **(E)** and GSEA-KEGG **(F)** analysis in GBM. GSEA-GO **(G)** and GSEA-KEGG **(H)** analysis in LGG.

In our preliminary analysis, we have determined that high expression of MTTP was associated with poor prognosis of OS in STAD, COAD, GBM and LGG. To elucidate the potential biological pathways regulated by MTTP, we conducted GSEA in these cancers. First, we drew the heat maps of the DEGs between MTTP high- and low-expression groups in these four cancers (**Supplementary Figure 7**). Then, we performed GSEA-GO and GSEA-KEGG analyses (**Supplementary Tables 1**, **2**). In STAD, GSEA-GO analyses showed lipid transport, cholesterol homeostasis and BMP signaling pathway. GSEA-KEGG analyses showed IL-17 signaling pathway, cGMP-PKG signaling pathway, PPAR signaling pathway, Toll-like receptor signaling pathway, TNF signaling pathway and neutrophil extracellular trap formation. In COAD, GSEA-GO analyses showed adaptive immune response, immune response-regulating cell surface receptor signaling pathway, lymphocyte mediated immunity and lipid transport. GSEA-KEGG analyses showed antigen processing and presentation, natural killer cell mediated cytotoxicity, TNF signaling pathway, NF-kappa B signaling pathway and Wnt signaling pathway. In GBM, GSEA-GO analyses showed canonical NF-kappaB signal transduction, fatty acid metabolic process and immunoglobulin mediated immune response. GSEA-KEGG analyses showed cell cycle, ferroptosis, gycolysis/gluconeogenesis, p53 signaling pathway, NF-kappaB signaling pathway and TNF signaling pathway. In LGG, GSEA-GO analyses showed antigen processing and presentation of peptide antigen, regulation of interleukin-8 production, negative regulation of T cell activation and canonical NF-kappaB signal transduction. GSEA-KEGG analyses showed oxidative phosphorylation, antigen processing and presentation, JAK-STAT signaling pathway and NF-kappa B signaling pathway. Meanwhile, we showed the top 5 GSEA-GO and GSEA-KEGG pathways in the four tumors (**Figure 7**). These mainly focused on the carcinogenic pathways (such as NF-kappaB signaling pathway, TNF signaling pathway), mechanism of immune regulation (such as antigen processing and presentation, adaptive and innate immune systems) and lipid metabolism (such as lipid transport, fatty acid metabolic process). Overall, these results indicated that MTTP plays a major role in tumor development and tumor immunity.

### 3.6 MTTP serves as a prognostic biomarker for GC

**Figure 8:**
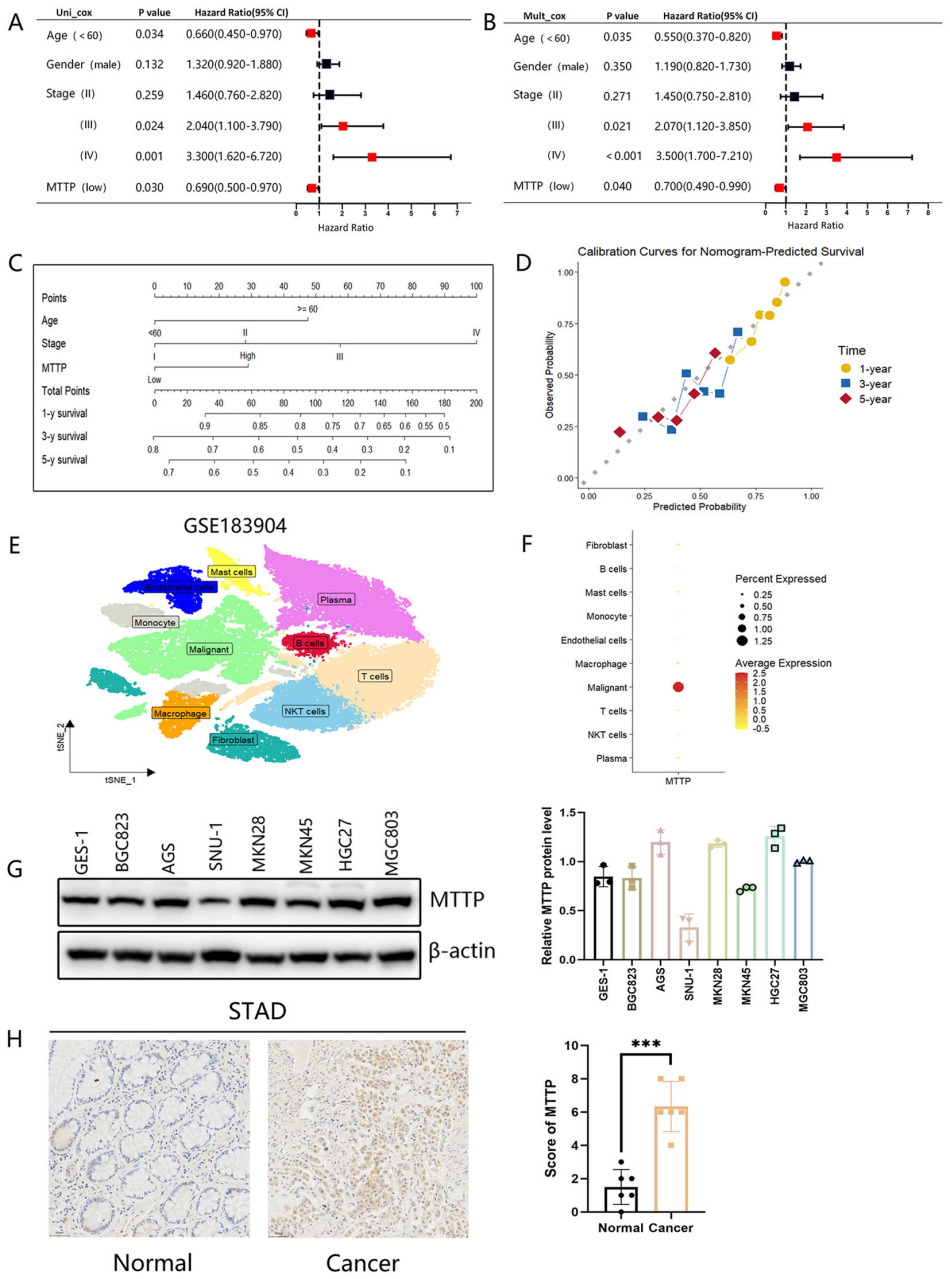
Analysis of the clinical relevance of MTTP in GC. Prognostic significance of MTTP in GC by univariate **(A)** and multifactorial COX analysis **(B)**. **(C)** Nomogram based on MTTP expression and clinical information. **(D)** The calibration curve for predicting 1-, 3-, and 5-year OS. **(E)** t-SNE plot demonstrating 10 clusters from 15 patients with GC (GSE183904). **(F)** Dot plot showing that MTTP was primarily expressed in malignant tumor cells. **(G)** The expression of MTTP protein was detected in seven GC cell lines compared with an immortalized gastric cell line (GES-1) and quantified using a gray scale analysis. **(H)** The representative IHC images of MTTP in GC and adjacent normal tissues ***, P <0.001.

The above results showed that MTTP was overexpressed in GC at the mRNA level, and survival analysis showed that the expression level of MTTP was closely correlated with OS (P = 0.0047) in patients with GC. Therefore, we explored the possible biological functions and mechanisms of MTTP in GC. Initially, we evaluated the prognostic value of MTTP in GC using univariate and multivariate COX regression models based on TCGA database, both of which suggested that MTTP was an independent prognostic marker (**Figures 8A, B**). Furthermore, we constructed a nomogram and its calibration curve based on the patients’ age, tumor stage and MTTP expression (**Figures 8C, D**). The analysis showed that the c-index of the nomogram was 0.70, indicating good prediction performance. scRNA-seq analysis of GC tumor tissues revealed that MTTP was primarily expressed in malignant tumor cells rather than other cell types (**Figures 8E, F**, **Supplementary Figure 8**). We further analyzed the protein expression of MTTP in GC cells, the result showed that MTTP expression was elevated in more than half of the 7 GC cells, compared with the GES-1 (**Figure 8G**). The immunohistochemical (IHC) staining result indicated that MTTP was overexpressed in GC (**Figure 8H**).

### 3.7 Effect of MTTP on the proliferation, migration, and invasion of GC

**Figure 9:**
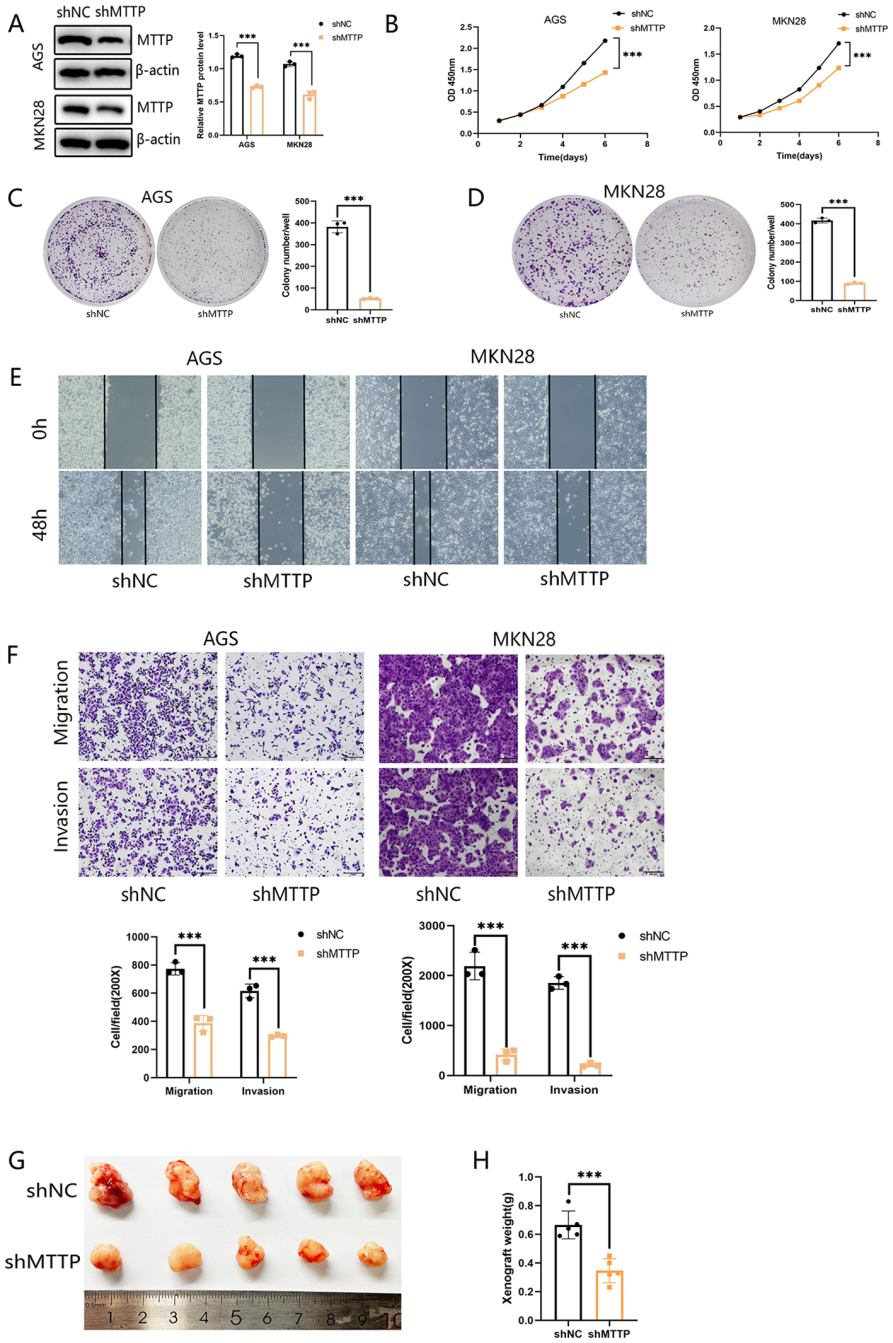
Knockdown of MTTP inhibited the proliferation, migration and invasion abilities of GC cells. **(A)** Validation of MTTP knockdown efficiency by western blotting after shRNA transfection of two gastric cancer cells and quantified using a gray scale analysis. **(B–D)** CCK8 and Foci formation showing that inhibition of MTTP reduced the proliferation. **(E)** Wound-healing assay showing that MTTP knockdown inhibited the migration of GC cells at 0 and 48h after scratch wounding. **(F)** Transwell assay showing that silencing MTTP expression effectively inhibited cell migration and invasion. **(G, H)** Representative images of the xenograft tumors formed in nude mice (n=5) and the weights of the xenograft tumors were measured and analyzed ***, P <0.001.

To investigate the biological function of MTTP in GC cells, we knocked down MTTP in AGS and MKN28 cells with short hairpin RNAs (shRNAs) (**Figure 9A**). CCK8 proliferation assay showed that MTTP knockdown significantly inhibited tumor cell growth rate (**Figure 9B**). Foci formation showed that MTTP knockdown significantly reduced colony frequency and size (**Figures 9C, D**). Wound healing and transwell assay demonstrated that cell migration and invasion were markedly decreased after MTTP knockdown (**Figures 9E, F**). To validate the effect of MTTP on tumor growth *in vivo*, subcutaneous xenograft tumor mouse models were established. MTTP knockdown significantly inhibited the tumor growth (**Figures 9G, H**).

### 3.8 Inhibition of MTTP conferred sensitivity to oxaliplatin by inducing ferroptosis in GC cells

**Figure 10:**
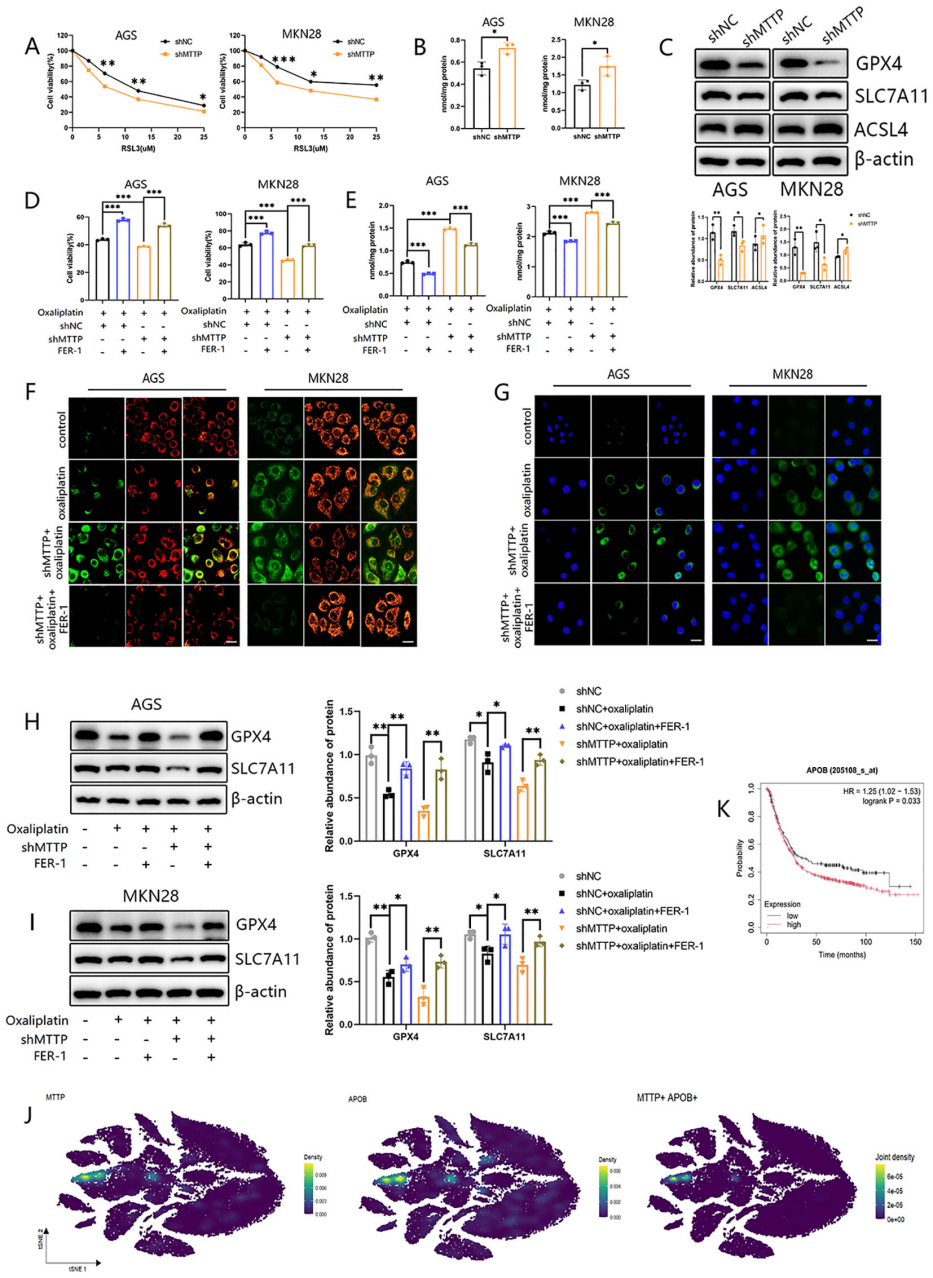
Knockdown of MTTP increased sensitivity to ferroptosis in GC cells. **(A)** The GC cells were exposed to ferroptosis inducer RSL3 for 24h and cell viability was measured by CCK8 assay. **(B)** The MDA level of GC cells. **(C)** WB revealed the expression levels of GPX4, SLC7A11 and ACSL4 in GC cells with MTTP knockdown and quantified using a gray scale analysis. **(D)** The GC cells were exposed to different drugs and cell viability was measured by CCK8 assay. **(E)** The GC cells were exposed to different drugs and the MDA level was measured. **(F)** JC-1 staining in GC cells (scale bar = 20 μm). **(G)** C11-BODIPY staining in GC cells (scale bar = 20 μm). **(H)** WB revealed the expression levels of GPX4 and SLC7A11 in AGS treated with different drugs and quantified using a gray scale analysis. **(I)** WB revealed the expression levels of GPX4 and SLC7A11 in MKN28 treated with different drugs and quantified using a gray scale analysis. **(J)** The expression profiles of MTTP and APOB in different cells in GC (GSE183904). **(K)** The overall survival curves of patients with GC stratified based on APOB expression in Kaplan Meier plotter web. *, P <0.05; **, P <0.01; ***, P <0.001.

Oxaliplatin exerts the chemotherapeutic effect in tumor by directly combining with DNA to cause DNA irreversible damage including mitochondrial DNA (mtDNA). Continuous mtDNA damage may leads to dysfunction of oxidative phosphorylation (OXPHOS) components, which in turn produces excess ROS and disrupts normal mitochondrial function (26, 27). Several study mentioned that oxaliplatin induced ferroptosis, the ROS-dependent cell death type, in colorectal cancer (20, 28), hepatocellular carcinoma (29) and GC (30). Therefore, we hypothesized that inhibition of MTTP could cause GC cells to be sensitive to oxaliplatin by inducing ferroptosis. Firstly, AGS and MKN28 cells with different expressions of MTTP were treated with ferroptosis inducer RSL3, and cell survival was determined. The results showed that MTTP knockdown significantly sensitized cells to RSL3-induced cell death (**Figure 10A**). Malondialdehyde (MDA), the product of lipid peroxidation, was increased in MTTP knockdown AGS and MKN28 cells (**Figure 10B**). Additionally, we evaluated the expression of ferroptosis marker genes(SLC7A11, GPX4 and ACSL4) in GC cells with different expressions of MTTP. MTTP knockdown in AGS and MKN28 cells showed the downregulation of GPX4 and SLC7A11 expression and upregulation of ACSL4 expression (**Figure 10C**). These results suggested that MTTP knockdown promoted ferroptosis of GC cells. Then, We treated GC cells with oxaliplatin for 48 hours, the results showed that oxaliplatin induced the decreased cell viability, while Ferrostatin-1(FER-1, 10 μM), the inhibitor of ferroptosis, reversed the inhibitive effect of oxaliplatin on cell viability of AGS and MKN28 cells partially. Compared with the control group, GC cells viability decreased more in the MTTP knockdown group(**Figure 10D**). More importantly, MTTP knockdown could induce ferroptosis, accompanying by the reduced expression of GPX4 and SLC7A11, the increased level of MDA, while FER-1 reversed the effect of MTTP knockdown in oxaliplatin-treated GC cells(**Figures 10E, H, I**). The ratio of red to green fluorescence in JC-1 dye-treated cells reflected the changes in the mitochondrial membrane potential. High concentrations of JC-1 aggregate normal mitochondria emit red fluorescence, which shifts to green fluorescence at low concentrations. After 48h of oxaliplatin treatment, the red/green fluorescence ratio decreased, indicating a reduction in the mitochondrial membrane potential. MTTP knockdown further reduced this ratio. However, the reduction was reversed by FER-1 (**Figure 10F**). C11-BODIPY staining also showed that lipid peroxidation was significantly enhanced in GC cells with MTTP knockdown in response to oxaliplatin stimulation and inhibited by FER-1 (**Figure 10G**). These results demonstrated that MTTP knockdown caused GC cells to be more sensitive to oxaliplatin induced ferroptosis.

MTTP can transfer lipids to APOB (31). scRNA-seq analysis revealed that cell clusters with elevated MTTP also exhibited high levels of APOB expression (**Figure 10J**). Survival analysis indicated that patients exhibiting high APOB expression had a poorer prognosis (**Figure 10K**), and APOB was reported to be related to cellular stress (32). Consequently, it was plausible to hypothesize that the inhibitory effect of MTTP on ferroptosis may be associated with APOB.

### 3.9 Macrophages may be involved in the process of ferroptosis regulated by MTTP

**Figure 11:**
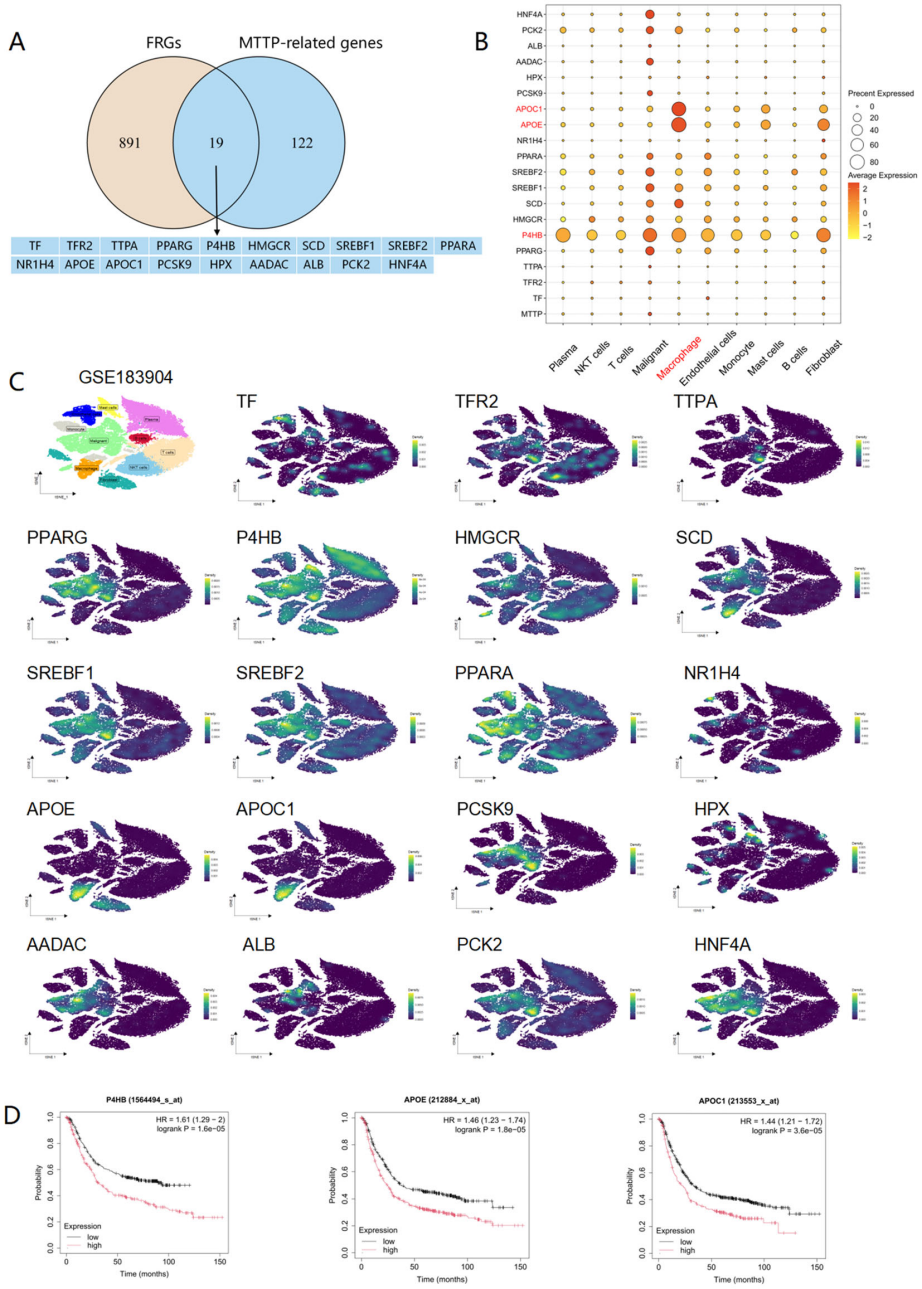
Expression profile of ferroptosis genes associated with MTTP at the single-cell level in GC (GSE183904). **(A)** An intersection analysis of FRGs and MTTP-related genes. **(B)** Dot plot of 19 genes in different cell cluster. **(C)** The expression profiles of 19 genes in different cells. **(D)** The overall survival curves of patients with GC stratified based on P4HB, APOE, APOC1 expression in Kaplan Meier plotter web.

Currently, there is only a single study that had explored the connection between MTTP and ferroptosis, this research proposed that exosomes carrying MTTP, secreted by adipocytes, inhibited ferroptosis in cancer cells by suppressing ZEB1 transcription. In order to uncover additional mechanisms, we obtained a total of 910 ferroptosis related genes (FRGs) from the FerrDbv2 website (http://www.zhounan.org/ferrdb/) (**Supplementary Table 4**). Then, we conducted an intersection analysis between FRGs and MTTP-related genes (100 MTTP-associated genes in GEPIA2 website and 50 MTTP-binding proteins in STRING website) and revealed 19 overlapping genes (**Figure 11A**). Leveraging the scRNA-seq of GC, we characterized the expression patterns of these 19 genes and observed that P4HB gene was highly expressed across all cell types, notably in malignant tumor cells, macrophages, and fibroblasts(**Figures 11B, C**). Furthermore, the APOE and APOC1 genes were prominently expressed in macrophages (**Figure 11B**). Survival analysis demonstrated that the P4HB, APOE, and APOC1 genes were significantly associated with the prognosis of GC patients(**Figure 11D**). Thus, we posited that macrophages might play a role in the process of ferroptosis regulated by MTTP.

## 4 Discussion

Increasing evidence shows that lipid metabolism is commonly enhanced at various stages of cancer progression. These metabolic shifts not only provide the necessary energy to power tumor cells but also initiate signaling pathways and epigenetic modifications. Moreover, they alter the composition of cellular membranes, which can facilitate the process of metastasis (33). On the other hand, the tumor-infiltrating immune cells that are also exposed to excess lipids can undergo lipid peroxidation, which impairs their function (34). MTTP is the major lipid transfer protein that transfers phospholipids and triacylglycerols to nascent APOB for the assembly of lipoproteins in hepatocytes and enterocytes (31, 35). Researchs had demonstrated that wrappER-mitochondria contact could regulate hepatic systemic lipid homeostasis. Disruptions in wrappER-mitochondria contact resulted in a reduction of VLDL secretion and an elevation of hepatic fatty acids, the acute liver-specific ablation of MTTP promoted the remodeling of wrappER-mitochondria contact (36). Furthermore, the ablation of MTTP triggered fatty acid β-oxidation in mitochondria and peroxisomes (37). Hence, MTTP can affect the operations of multiple intracellular organelles that partake in both the synthesis and degradation of fatty acids, with this impact potentially mediated by the secretion levels of VLDL. In antigen presenting cells, MTTP promoted the proper lipidation of CD1 proteins (12), CD1d is a major histocompatibility complex (MHC) class I–related molecule that functions in glycolipid antigen presentation (38, 39). In addition, MTTP regulated the lipolysis of adipocytes by inhibiting ATGL activity, which was independent of its lipid transfer capability (40). Beyond its potent physiological role in normal tissues, MTTP have been demonstrated to be associated with the development and progression of several tumors, such as glioblastoma (22), liver cancer (41) and colorectal cancer (20).

We performed an analysis of MTTP expression in pan-cancer using the TCGA and GTEx. Our findings revealed that the mRNA level of MTTP was significantly upregulated in 10 cancer types and downregulated in 12 types. We also analyzed the relationship between MTTP expression and clinical staging, MTTP declined as the clinical stage progresses in BRCA, whereas its expression escalated with advancing clinical stages in KIRC and KIRP. Subsequently, we examined the relationship between MTTP and clinical prognosis in different types of cancer patients. We found that high expression of MTTP predicted poorer OS in COAD, GBM, LGG, STAD, Myeloma and AML, concurrently, high expression of MTTP predicted poorer PFS in COAD, THCA, Myeloma and LUAD. In contrast, increased MTTP expression was linked to better OS for OV and better RFS for OV and BRCA. To date, only two studies have elucidated the mechanism by which MTTP promoted malignant tumor progression. Zhang et al. explored the oncogenic mechanism of MTTP in COAD, and found that the MTTP/PRAP1 complex enhanced the expression of GPX4 and xCT by suppressing ZEB1, which in turn limited the accumulation of polyunsaturated fatty acids and diminished ferroptosis in tumor cells (20). Besides, the role of MTTP in GBM was also explored by Yu et al., who concluded that EGFR-A289I variants upregulated MTTP, and inhibited the accumulation of tumor triacylglycerides, promoting gliomagenesis (22). These studies suggest that MTTP may play a pro-tumorigenic role by inhibiting the accumulation of excess lipids to prevent lipotoxicity induced cellular damage. With the exception of COAD and GBM, the prognostic implications of MTTP in tumors have not been previously reported. Our study is the first to identify the prognostic significance of MTTP across a range of cancers.

The tumor microenvironment (TME) is a dynamic and intricate ecosystem that surrounds and interacts with tumor cells and plays a pivotal role in the dissemination of cancer (42). However, there are no reports on MTTP expression and immune infiltration in tumors currently. We conducted an analysis of the correlation between MTTP and a spectrum of immune-related genes across various tumor types, including immune stimulators, immune inhibitors, chemokines, and their receptors. Notably, we observed positive correlations between MTTP expression and HHLA2 and CD160 in the majority of cancer types. Human endogenous retrovirus H long terminal repeat-associating protein 2 (HHLA2) is an immune-regulatory ligand and the newest member of B7/CD28 family (43). HHLA2 serves as an alternative checkpoint that is highly expressed across various solid tumor types. It plays dual roles in immune regulation, capable of exerting both immunostimulatory and immunosuppressive effects on CD4^+^ and CD8^+^ T cell (43, 44). Multiple studies have shown that high HHLA2 expression was associated with poor prognosis in triple-negative breast cancer (TNBC), HCC and clear cell renal cell carcinoma (ccRCC), but high level of HHLA2 was indicative of favorable outcomes in GBM (45). CD160 is a glycosylphosphatidylinositol (GPI)-anchored glycoprotein found on the cell surface of cytotoxic natural killer (NK) cells and specific T-cell subsets. It plays a crucial role in activating NK-cell cytotoxicity and cytokine production (46). Two researchs have shown that reduced CD160 expression was associated with compromised NK cell functionality and poor clinical outcomes in HCC and LUAD (47, 48). Comprising a variety of cellular and acellular components, the TME includes cancer cells, stromal cells (such as fibroblasts and mesenchymal stem cells), immune cells (such as T cells, B cells, macrophages, and dendritic cells), the extracellular matrix (ECM) and a multitude of signaling molecules (49). These elements are not isolated, rather they engage in ongoing and complex interactions that significantly influence tumor behavior. Lipid metabolic reprogramming is one of the mechanisms that alter the metabolic patterns of tumor and immune cells to meet their developmental needs and adapt to the complex TME (50). In this study, we found that MTTP expression was positively correlated with CD8^+^ T cell infiltration in most tumors, such as BRCA, CESC, HNSC-HPV+, KIRC, LUAD, SKCM and THCA. The correlation between MTTP expression and the infiltration of CAFs and M2 macrophages varied across different types of cancer. Single-cell analysis demonstrated distinct expression patterns of MTTP across immune cell subtypes, with predominant specific expression observed in central nervous system-resident macrophages(microglia) and secondary expression in bone marrow-derived macrophages. The pro-tumorigenic role of MTTP in GBM had been established, where it facilitated GBM development by modulating tumor triacylglycerides (22). Our study proposed a novel mechanism, suggesting that MTTP additionally influenced tumor cell progression through functional modulation of microglia in GBM.

We integrated the information on MTTP-interacting proteins and MTTP expression- associated genes across all tumors for a series of enrichment analyses and identified the potential impact of cholesterol metabolism, PPAR signaling pathway, Fat digestion and absorption, AMPK signaling pathway and insulin resistance in the pathogenesis of cancers. Meanwhile, we performed GSEA enrichment analysis in four tumor types, the results suggested that MTTP was associated with many carcinogenic pathways (such as NF-kappaB signaling pathway, TNF signaling pathway), pathways of immune regulation (such as antigen processing and presentation, adaptive and innate immune systems) and lipid metabolism (such as lipid transport, fatty acid metabolic process). Brozovic et al. (51) showed that hepatocytes and intestinal epithelial cells (IECs) from animals with conditionally deleted MTTP exhibited a diminished expression of CD1 proteins on the cell surface, which influenced the activation of invariant natural killer T (iNKT) cells, a key component of the immune response. Knockdown of MTTP in the human monocyte cell line U937 also led to a reduction in antigen presentation by CD1d to NKT cells (14). However, our pan-cancer analysis revealed a negative correlation between MTTP and NKT cells in tumor, suggesting that MTTP may exert distinct functional roles in tumor cells versus immune cells within the TME.

Our study is the first to show that MTTP can be used as a prognostic marker in patients with GC. We found that MTTP was elevated in GC and mainly expressed in malignant epithelial cells. MTTP promoted tumor proliferation, migration and invasion ability of cancer cells. Triphenyl phosphate (TPhP), a widely utilized organophosphate flame retardant, has become pervasively detected in various environmental matrices due to its extensive application in electronic products, construction materials, and industrial manufacturing processes (52). TPhP promoted the proliferation and migration of GC cells and had a high binding affinity with MTTP in molecular docking (53). Naringenin has been reported to inhibit the MTTP/APOB axis to suppress the progression of intestinal metaplasia (54). Additionally, knockdown of MTTP increased the sensitivity to ferroptosis in GC cells in our study. These results suggest that MTTP may be an effective target for the treatment of GC.

Within the TME, non-malignant cellular components including immune cells and stromal elements exert critical regulatory influences on neoplastic progression and ferroptotic susceptibility. Specifically, both tumor-associated macrophages(TAMs) and cancer-associated fibroblasts(CAFs) can adapt to ferroptosis through their intrinsic metabolic reprogramming, they may also influence ferroptosis in tumor cells via a paracrineal pathway (55–57). We identified 19 genes associated with ferroptosis among the MTTP-related genes, which were predominantly enriched in malignant epithelial cells, macrophages, and fibroblasts. Notably, the genes P4HB, APOE, and APOC1 stood out as the most prominent. APOC1 and APOE can be co-expressed in macrophages. Prolyl 4-hydroxylase subunit beta (P4HB) encodes the beta subunit of prolyl 4-hydroxylase, which is responsible for hydroxylating prolylresidues in preprocollagen. P4HB is an essential component of ER and is implicated in various cellular stress responses such as mitochondrial function damage, oxidative and endoplasmic reticulum stress (58, 59). Current evidences have indicated that P4HB could act as an oncogene, and its expression was notably associated with immune regulatory genes in certain types of cancer (60). Our previous studies have confirmed that APOC1^+^APOE^+^ macrophages participated in the establishment of pre-metastasis niche and promoted the formation of immunosuppressive TME (61). Inhibition of APOC1 promoted M2 TAMs to repolarize into M1 macrophages through the ferroptosis pathway in HCC (62), and exosomes derived from M2 TAMs that carry APOC1 could enhance the resistance to ferroptosis in osteosarcoma (63). Therefore, the inhibitory effect of MTTP on ferroptosis may be related to P4HB and APOC1^+^APOE^+^macrophages in GC.

Nonetheless, this study also has certain limitations. First, our study was mainly based on public databases, discrepancies arising from various analytical methods could yield divergent results. Second, we had no further experiments to verify the relationship between macrophages and MTTP in GC. Third, we only analyzed the mRNA and protein expression of MTTP in pan-cancer, lacking relevant data of MTTP enzyme activity in tumor tissues.

## 5 Conclusions

We investigated the differential expression of MTTP in cancer and found that it had prognostic value in multiple tumors. MTTP expression showed significant association with the infiltration of immune cells, and MTTP could be specifically expressed in microglia. *In vivo* and *in vitro* experiments confirmed that MTTP could promote the progression of GC and inhibit ferroptosis of GC cells. It is worth noting that macrophages may participate in the process of ferroptosis regulated by MTTP. Our study revealed the role of MTTP in tumors from the perspective of pan-cancer, providing a potential target for tumor therapy.

## Data Availability

The original contributions presented in the study are included in the article/**Supplementary Material**. Further inquiries can be directed to the corresponding authors.
