# Supplementary material for: Pan-cancer analysis reveals MTTP as a prognostic and immunotherapeutic biomarker in human tumors
Source: Front Immunol. 2025 Mar 27;16:1549965. doi: 10.3389/fimmu.2025.1549965 (PMC11983653; doi:10.3389/fimmu.2025.1549965)
Supplement: Supplementary file 1 [file DataSheet1.doc]

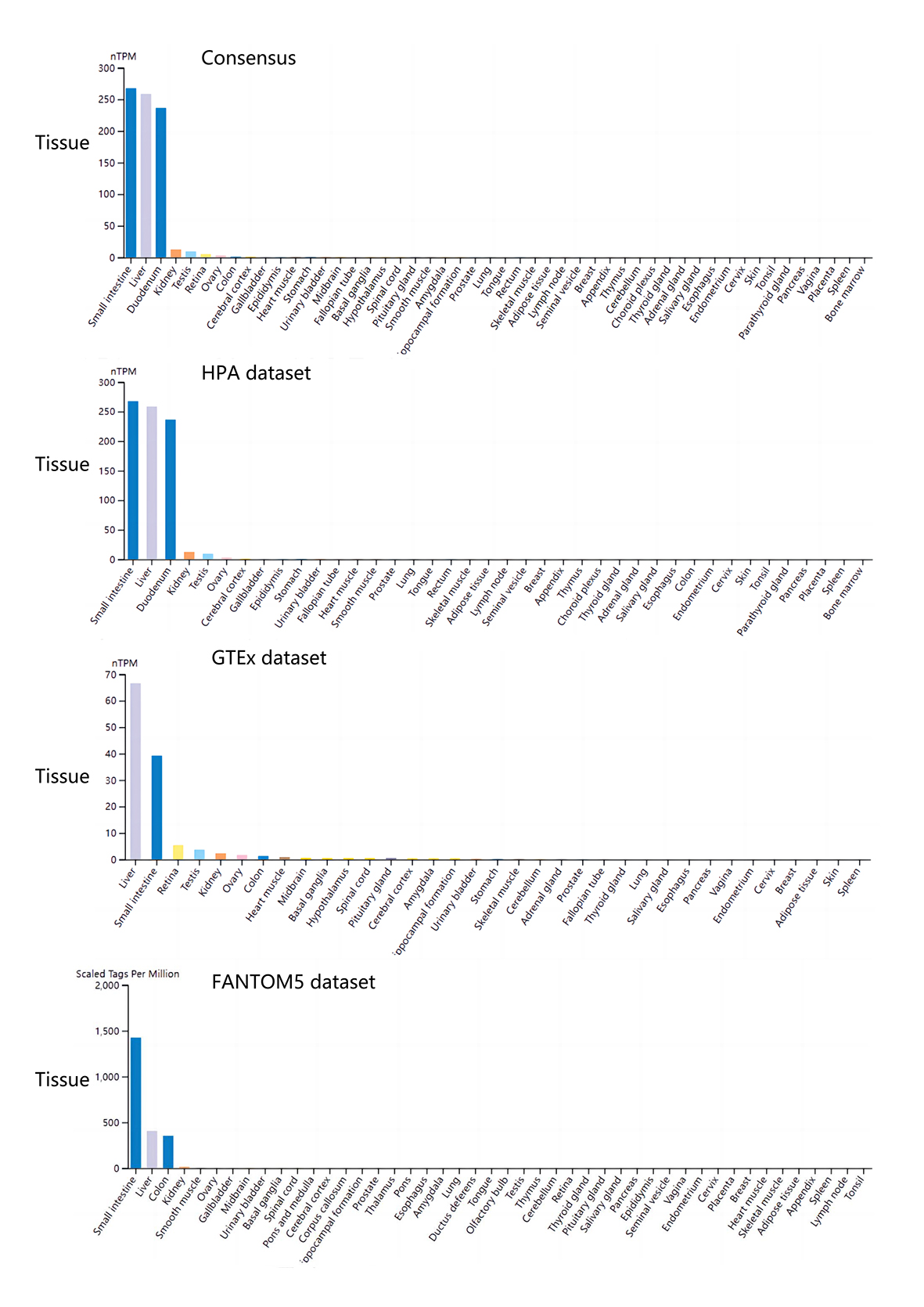


**Supplementary Figure 1:** **The expression level of MTTP in normal tissues based on Consensus, HPA, GTEx, and FANTOM5 dataset.**


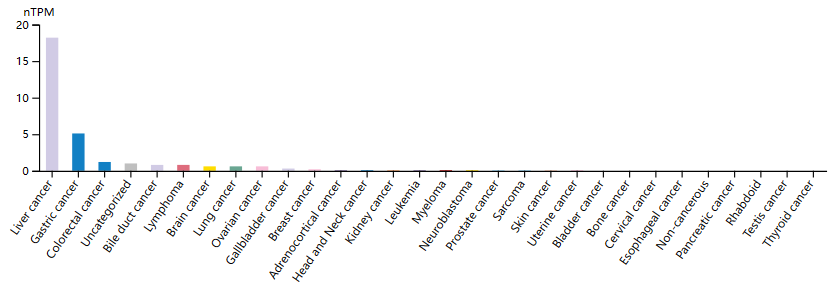


**Supplementary Figure 2: The expression of MTTP in different tumor cells based the HPA database.**


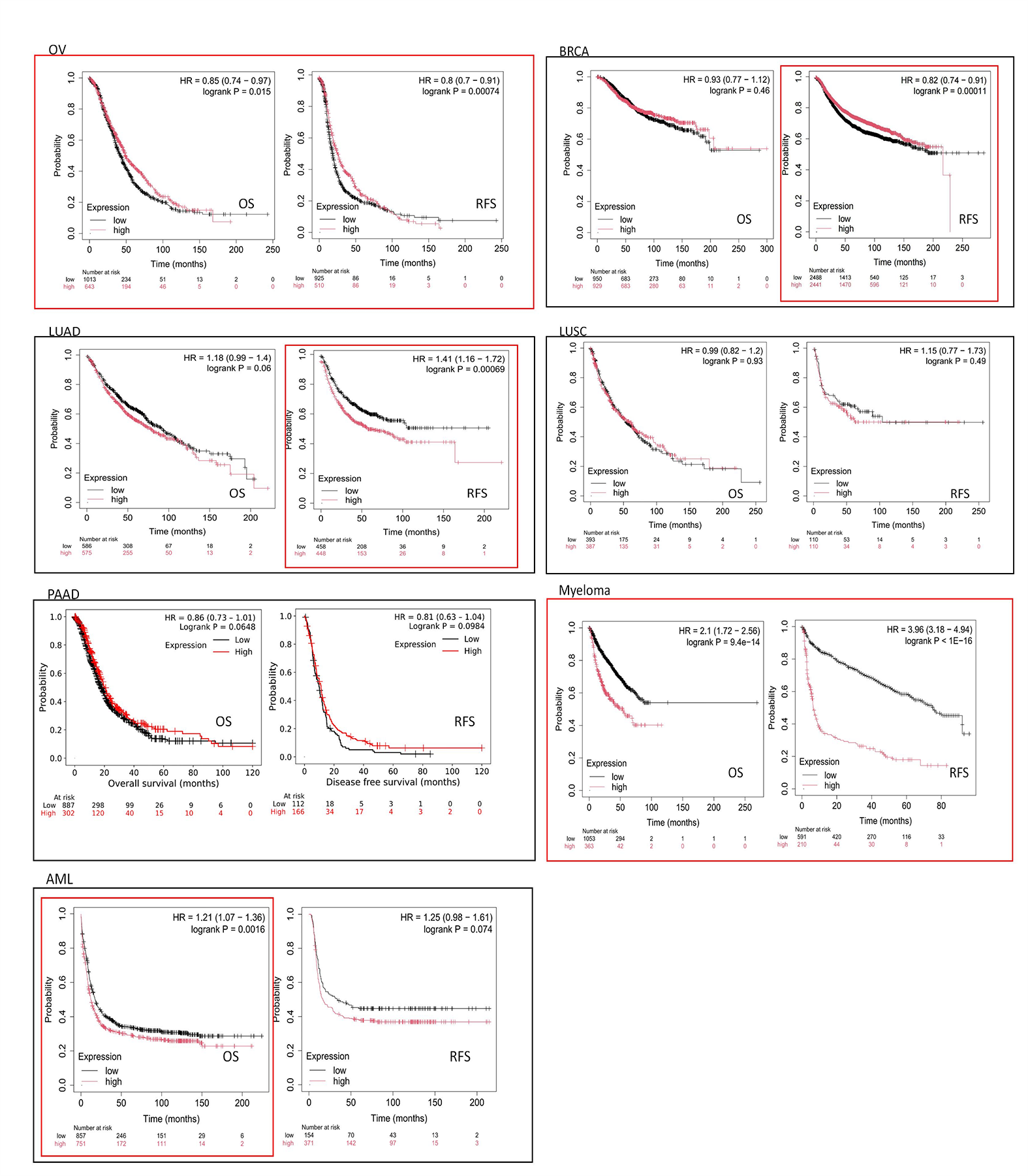


**Supplementary Figure 3:The Kaplan-Meier plotter was used to perform the survival analyses (OS and RFS) in OV, BRCA, LUAD, LUSC, PAAD, Myeloma and AML.** (The Kaplan-Meier plotter surrounded by red boxes suggest statistical significance)


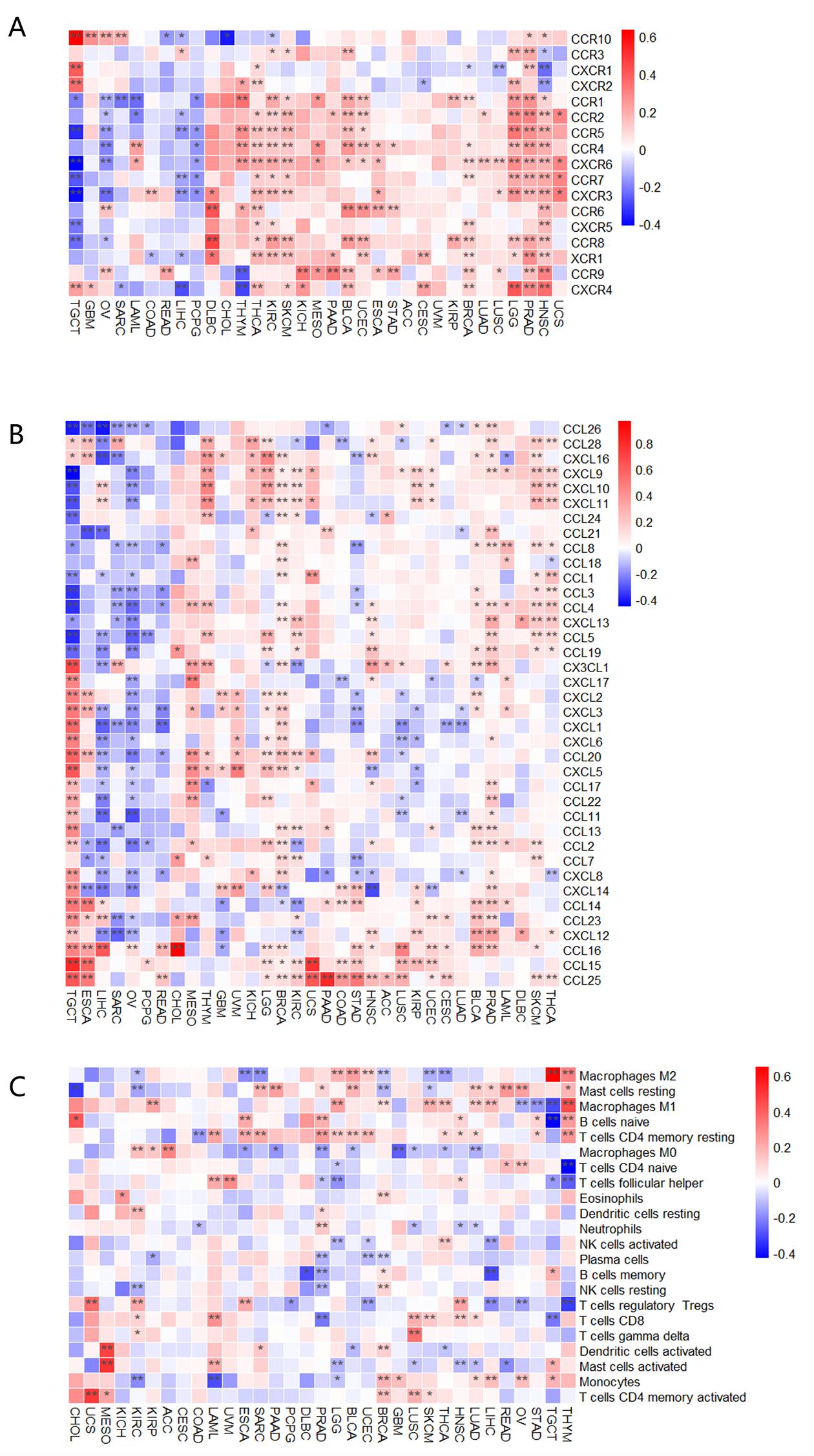


**Supplementary Figure 4:** **Correlations between MTTP expression and immune infiltration.** (A) Correlations between MTTP expression and chemokine receptors in different tumors. (B) Correlations between MTTP expression and chemokines in different tumors. (C) Correlations between MTTP expression and immune cells in different tumors.


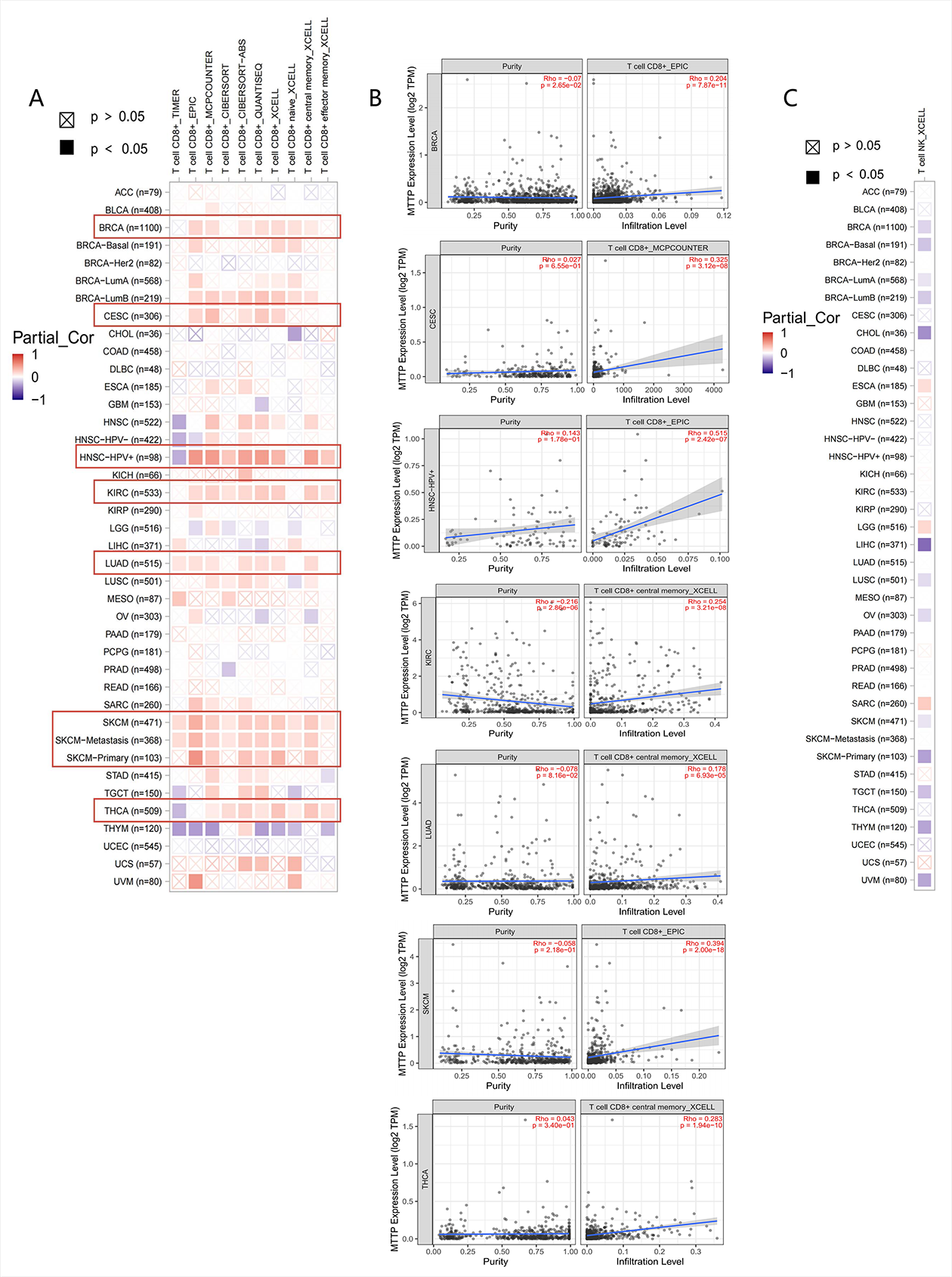


**Supplementary Figure 5: Correlations between MTTP expression and two types of immune cells infiltration.** (A) Correlation of MTTP expression in TCGA tumors with CD8^+^ T cell based The TIMER2.0. (B) Scatter plots of correlation with statistical differences in tumor. (C) Correlation of MTTP expression in TCGA tumors with NKT cell based The TIMER2.0.


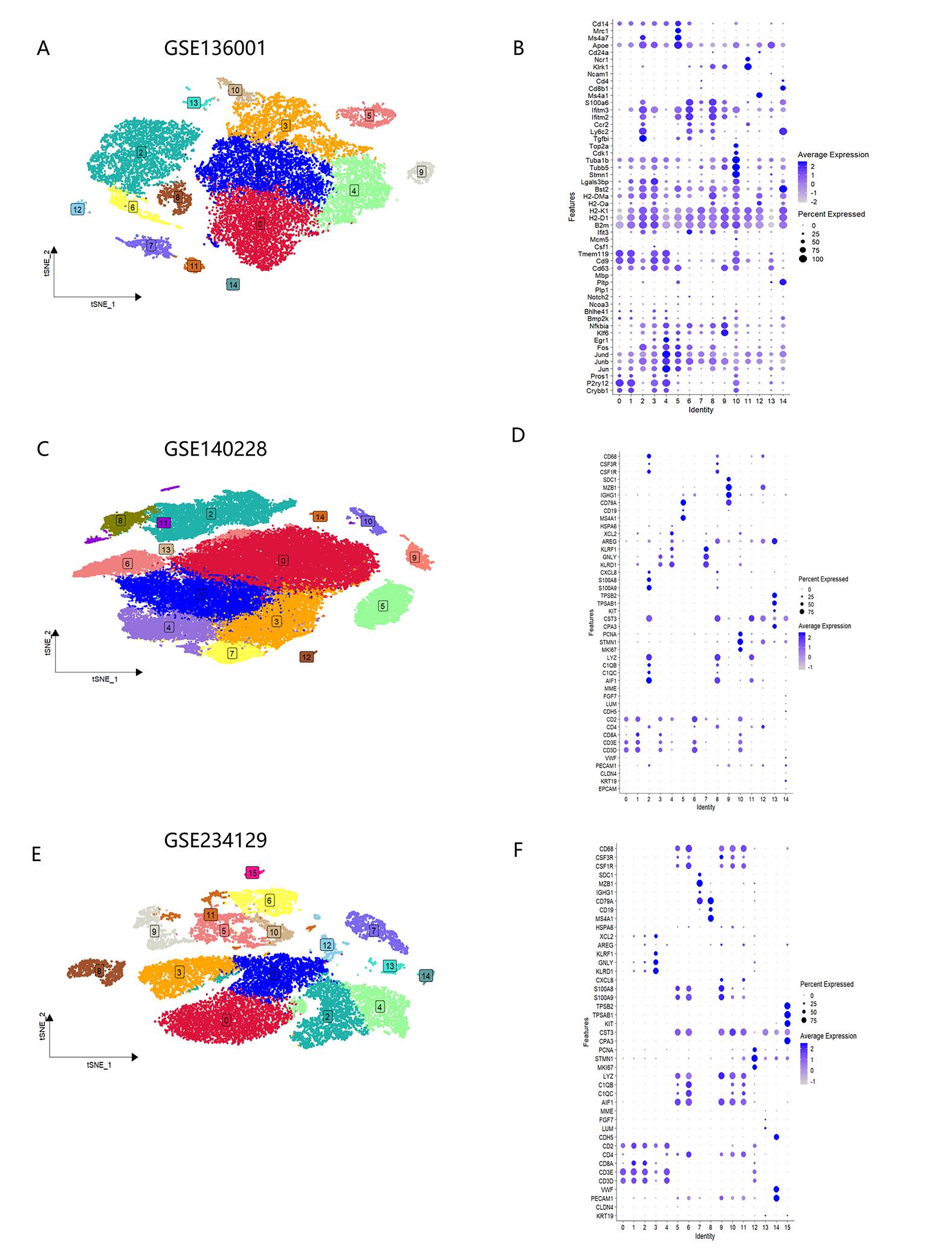


**Supplementary Figure 6: The distribution of cell subgroups in three types of tumor.** (A,B) 14 cell clusters in t-SNE and expression of cell maker genes in individual cell clusters in GBM(GSE136001). (C,D) 14 cell clusters in t-SNE and expression of cell maker genes in individual cell clusters in HCC(GSE140228). (E,F) 15 cell clusters in t-SNE and expression of cell maker genes in individual cell clusters in STAD(GSE234129).

**
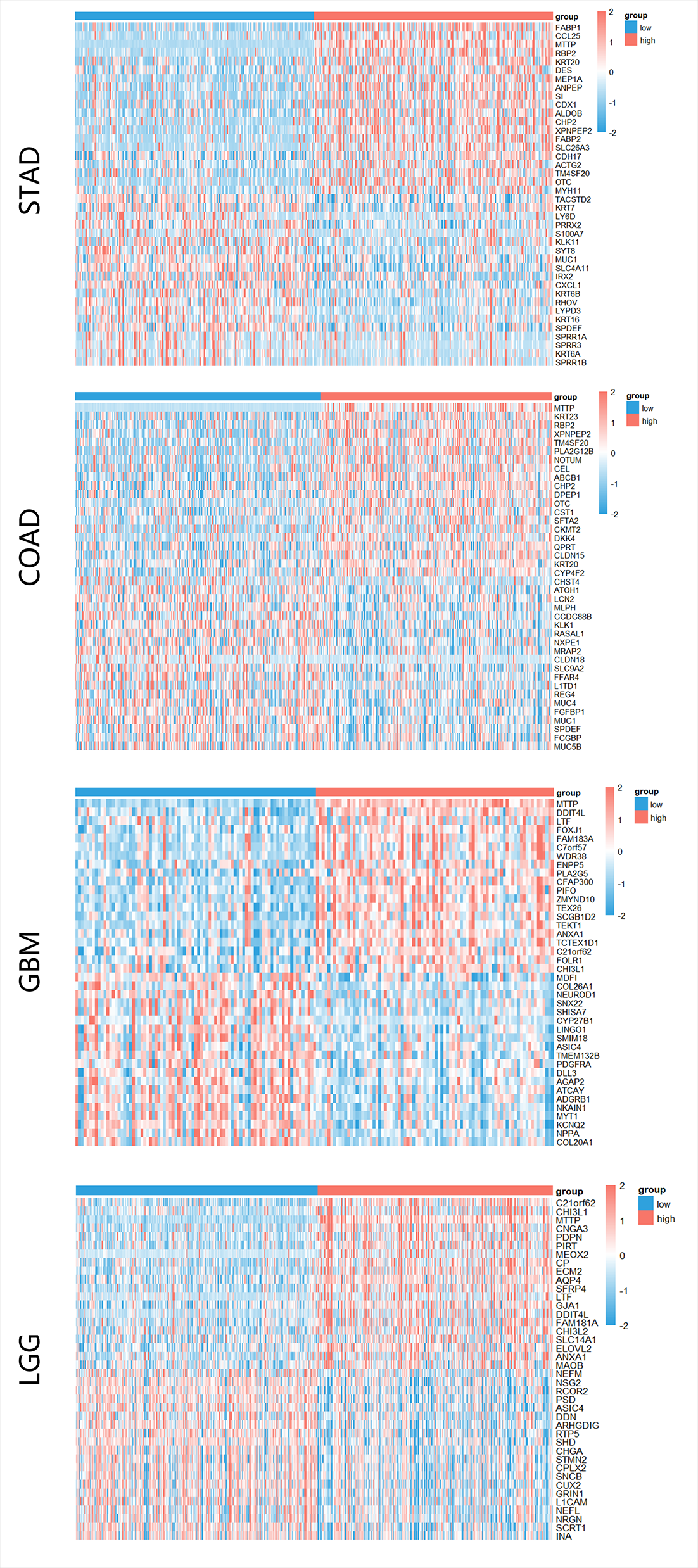
**

**Supplementary Figure 7:** **Heatmap of DEGs between MTTP high- and low-expression groups in STAD, COAD, GBM and LGG.**

**
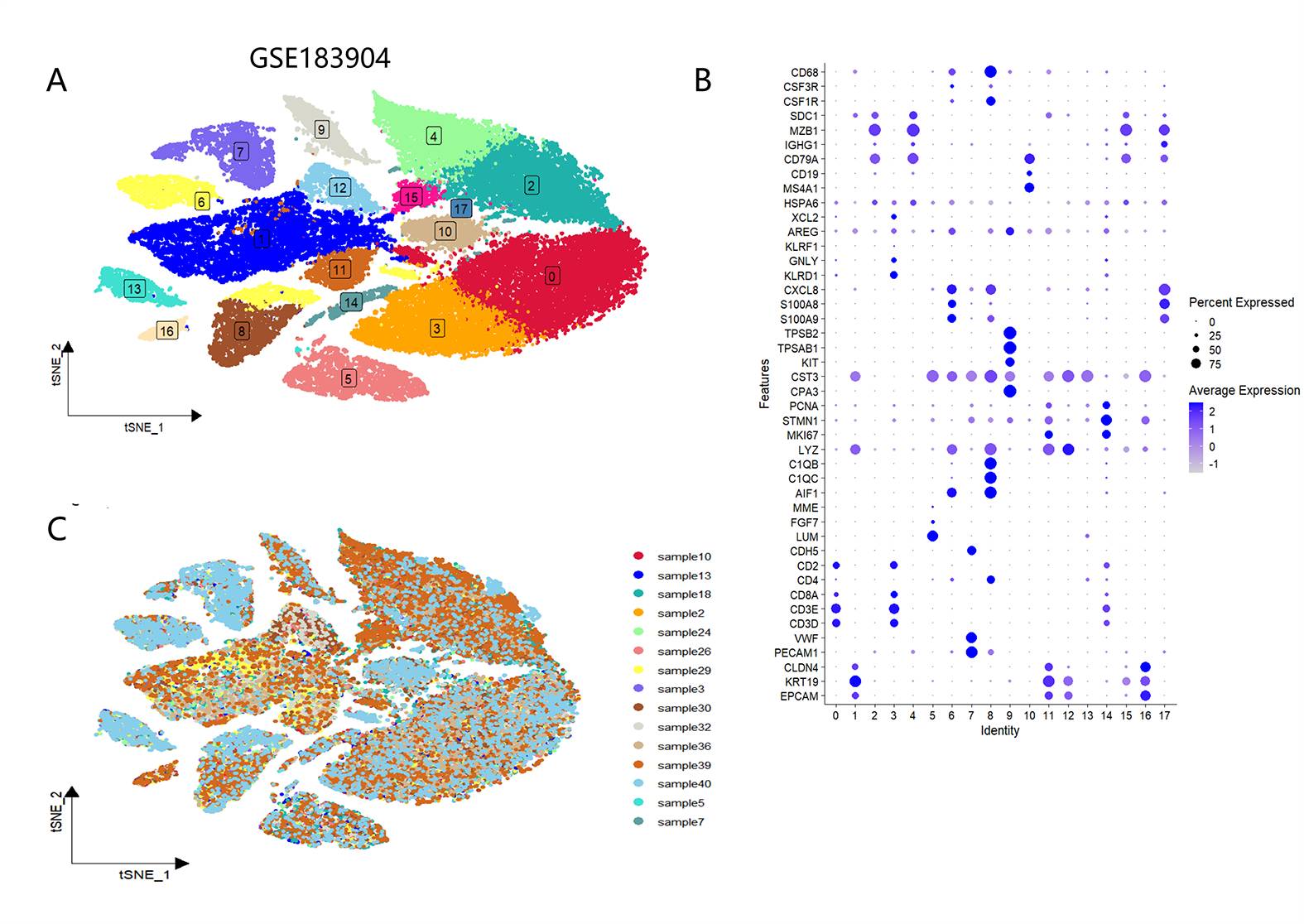
**

**Supplementary Figure 8: The distribution of cell subgroups and patient samples in GC.** (A) t-SNE shows that the total cells are divided into 17 clusters of GSE183904. (B) Expression of cell maker genes in individual cell clusters. (C) t-SNE shows the distribution of patient samples.
